# Supplementary material for: Design, Synthesis, Antifungal Activity, and Molecular Docking of Streptochlorin Derivatives Containing the Nitrile Group
Source: Mar Drugs. 2023 Jan 31;21(2):103. doi: 10.3390/md21020103 (PMC9958711; doi:10.3390/md21020103)

Design, synthesis, antifungal activity and molecular docking of streptochlorin derivatives containing nitrile group

Jing-Rui Liu[1], Ya Gao[1], Bing Jin[1], Dale Guo[2], Fang Deng[2], Qiang Bian[3], Haifeng Zhang[4], Xinya Han[5], Abdallah S. Ali[6], Ming-Zhi Zhang[1]*, Wei-Hua Zhang[1] and Yu-Cheng Gu[7]

[1] *Jiangsu Key Laboratory of Pesticide Science, College of Sciences, Nanjing Agricultural University, Nanjing 210095, China*

[2] *State Key Laboratory Breeding Base of Systematic Research Development and Utilization of Chinese Medicine Resources, School of Pharmacy, Chengdu University of Traditional Chinese Medicine, Chengdu, 611137, China*

[3] *National Pesticide Engineering Research Center (Tianjin), College of Chemistry, Nankai University, Tianjin 300071, China*

[4] *Department of Plant Pathology, College of Plant Protection, Nanjing Agricultural University, Nanjing 210095, China*

[5] *School of Chemistry & Chemical Engineering, Anhui University of Technology, Ma'anshan 243002, China*

[6] *Department of Microbiology, Faculty of Agriculture, Cairo University, Giza 12613, Egypt*

[7] *Syngenta Jealott's Hill International Research Centre, Bracknell RG42 6EY, Berkshire, UK*

1. ^{1}H NMR

Compound 2

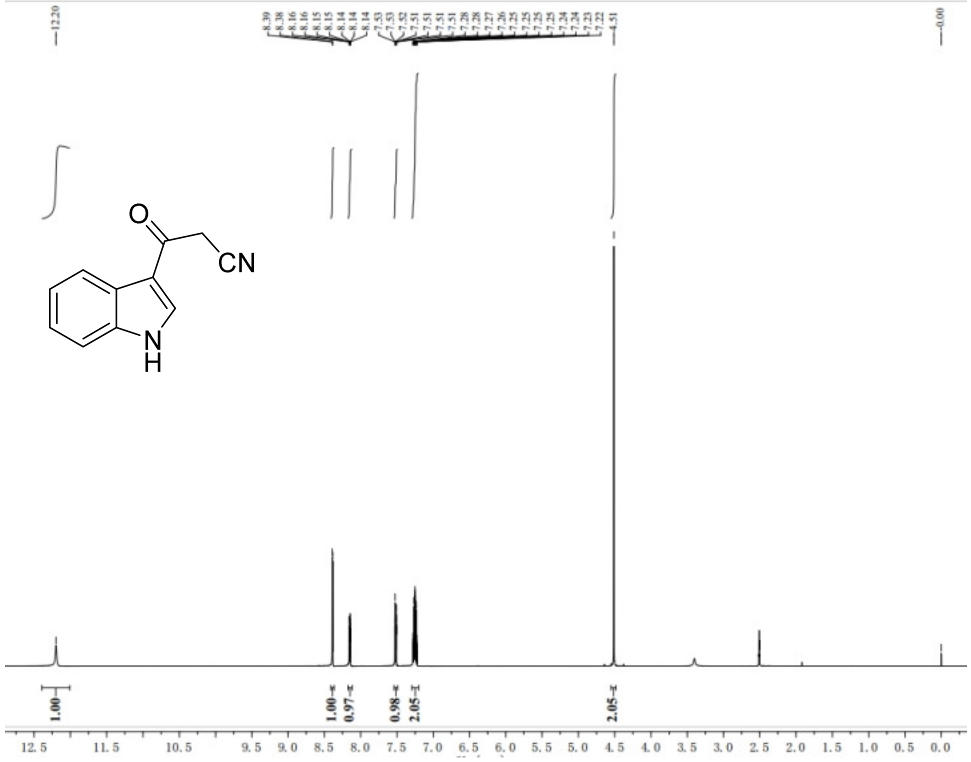

Compound 3a

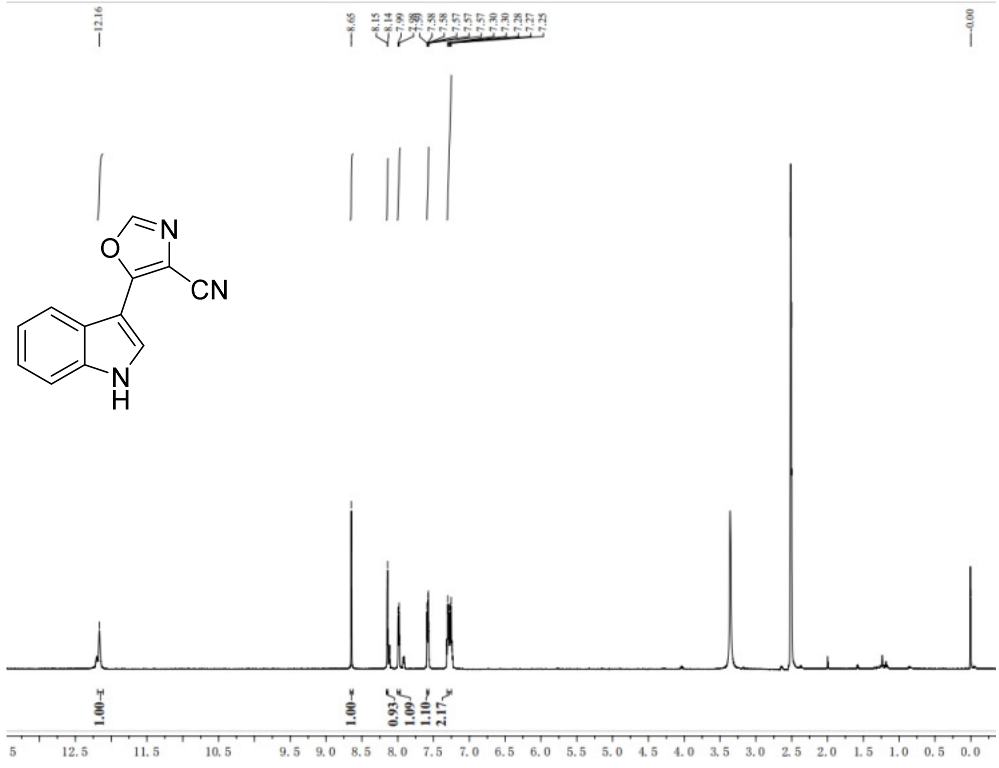

Compound 3b

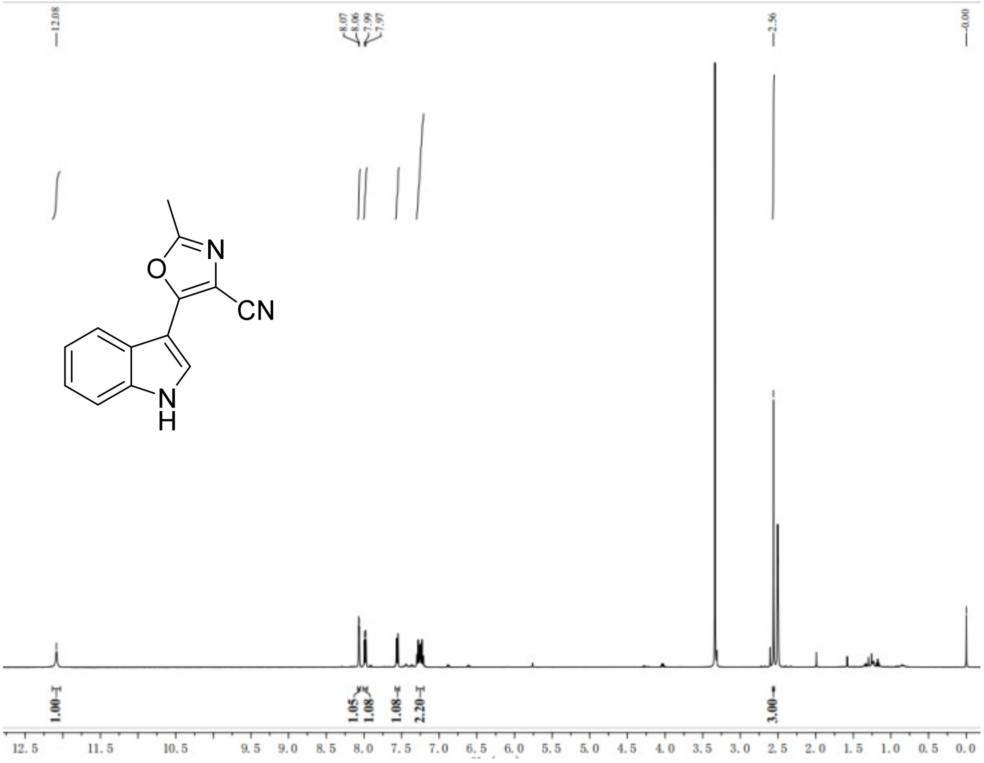

Compound 3c

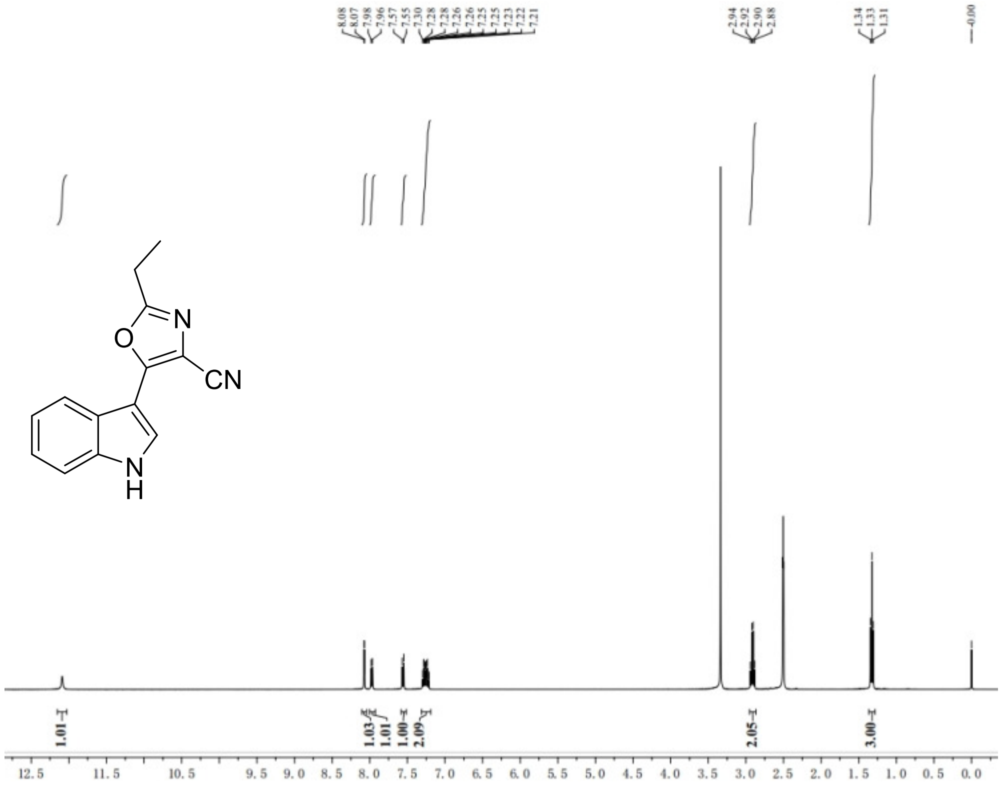

Compound 3d

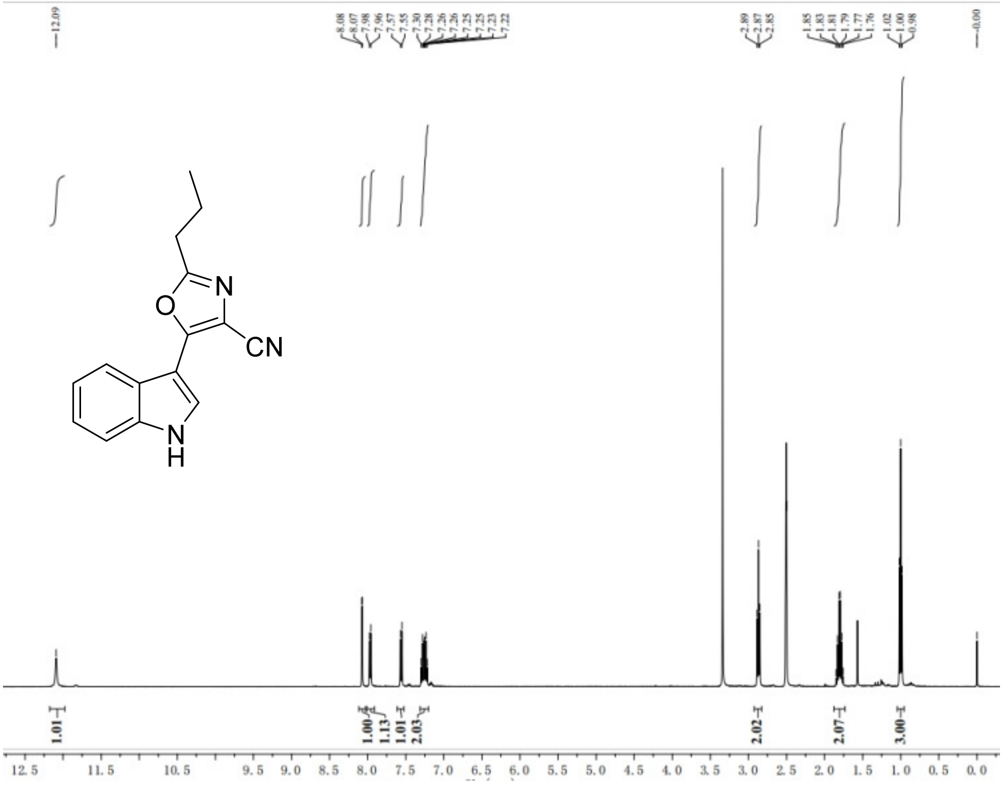

Compound 3e

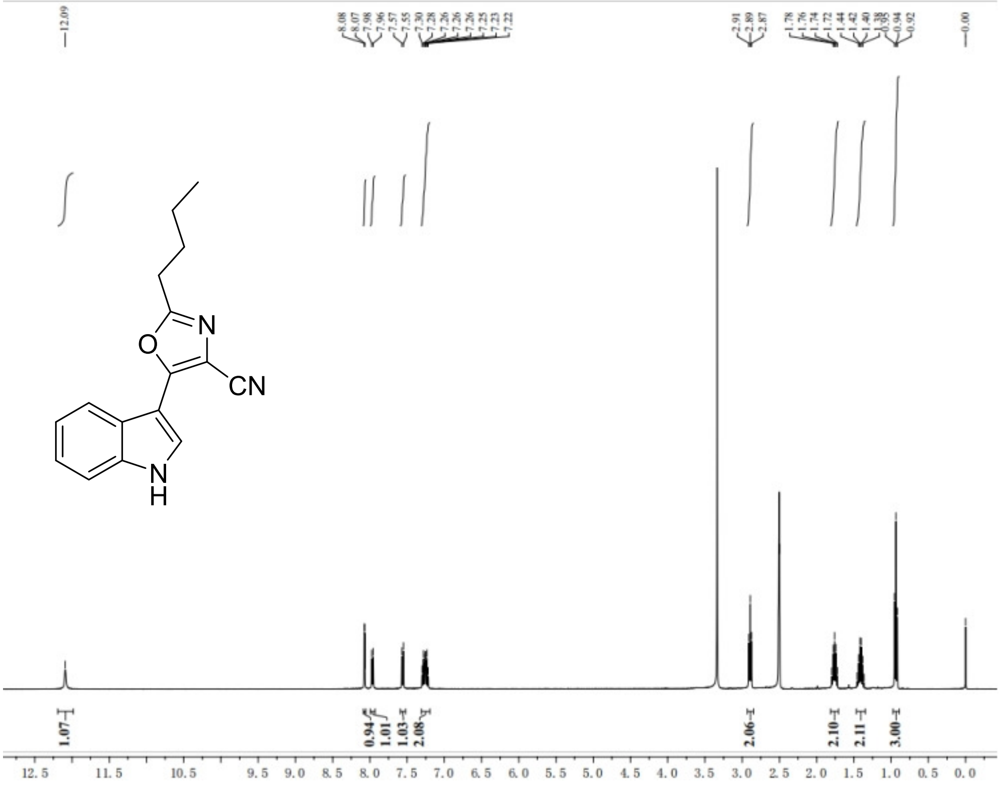

Compound 3f

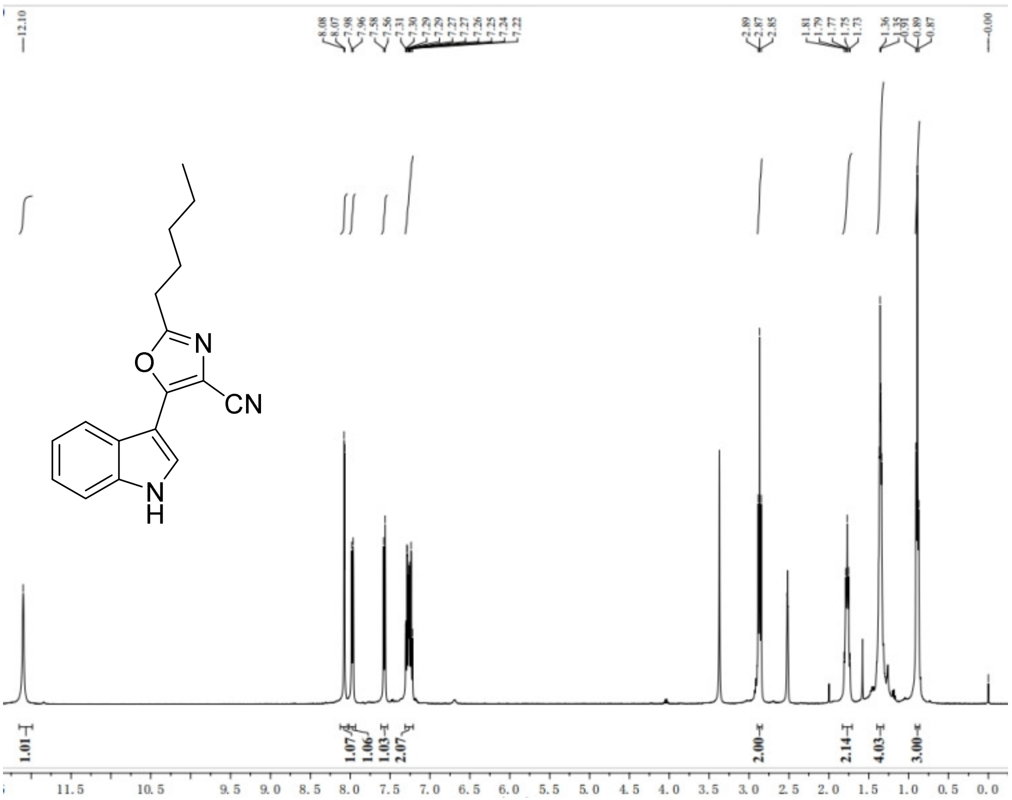

Compound 3g

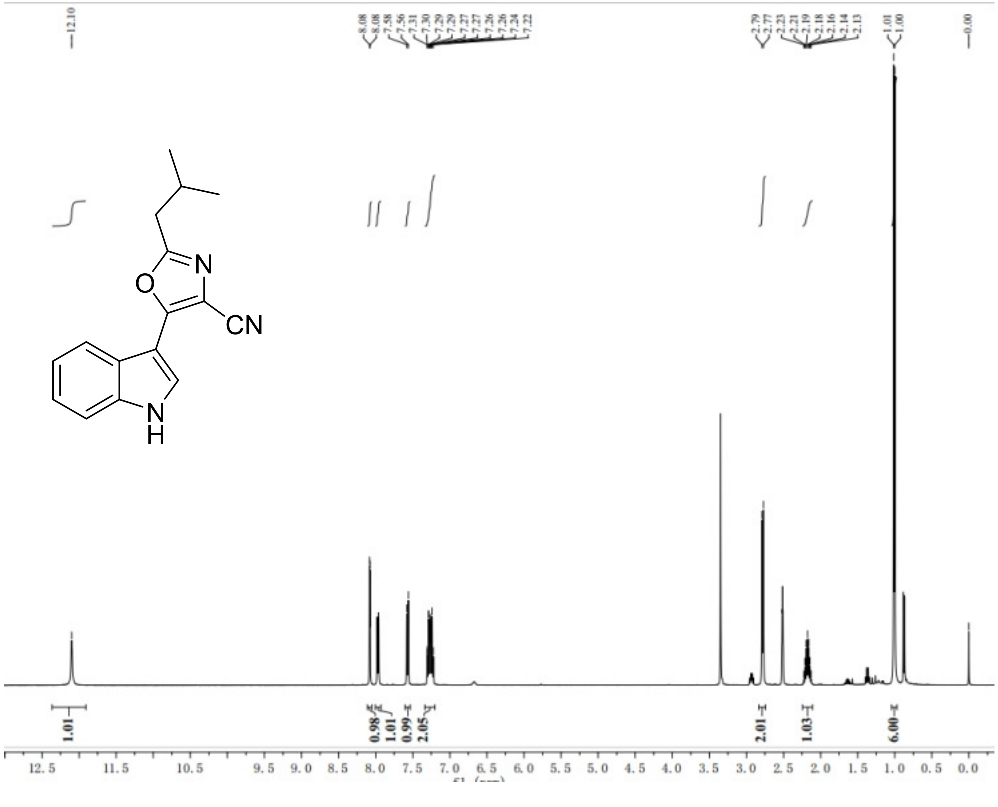

Compound 3h

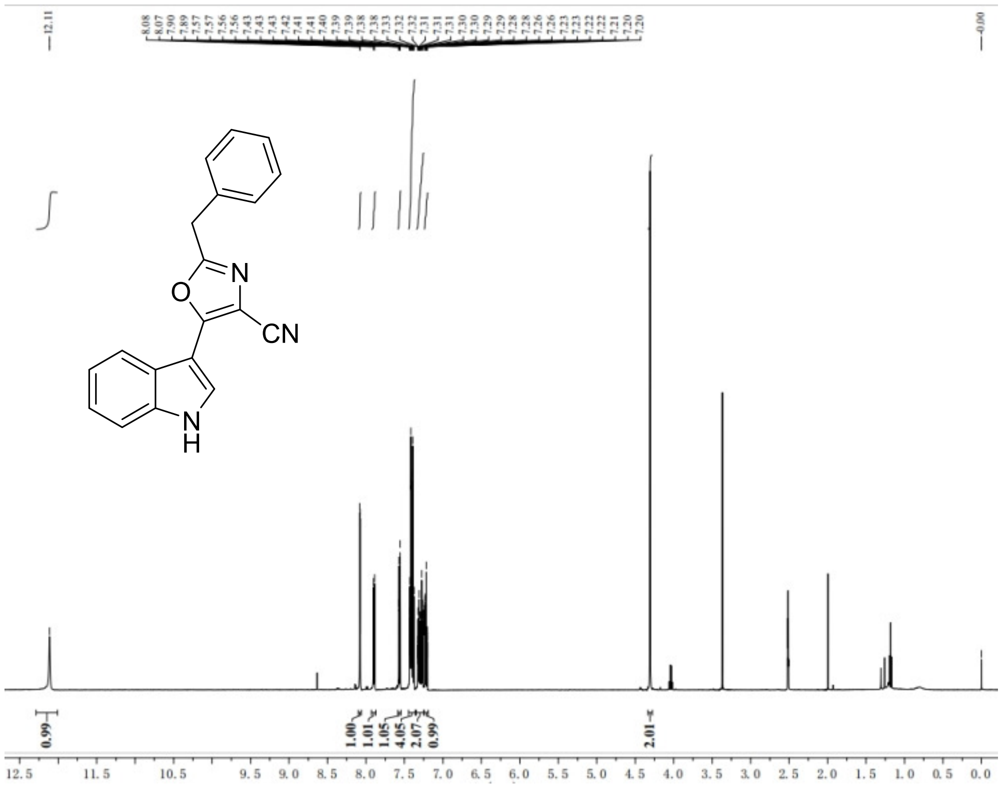

Compound 3i

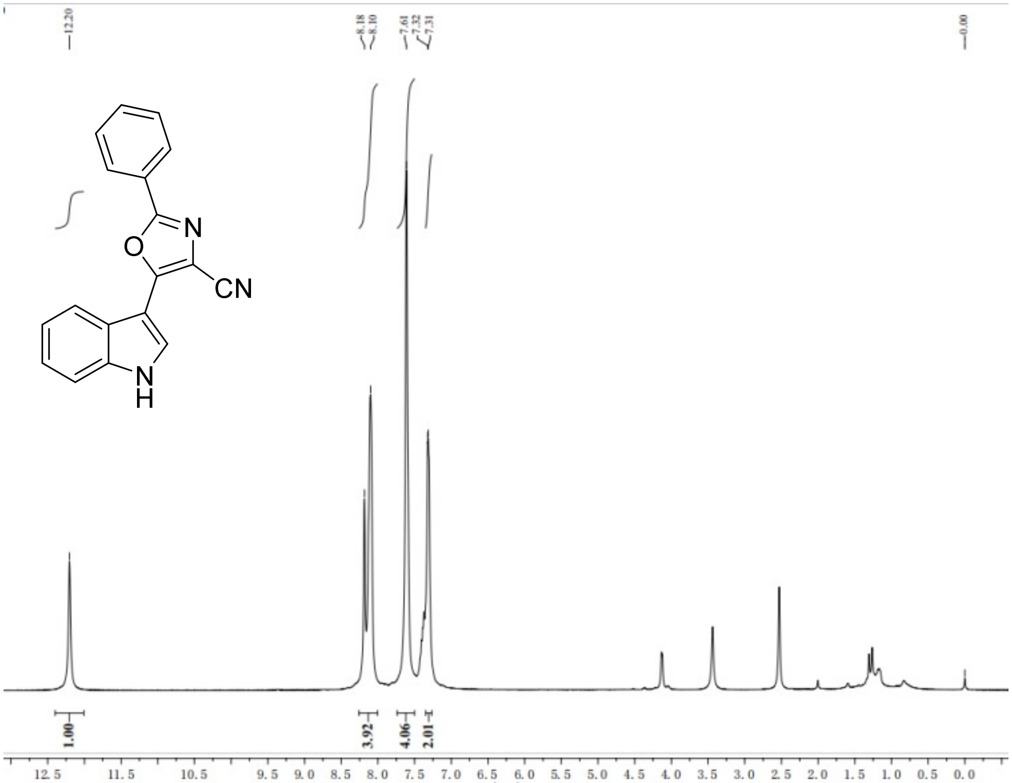

Compound 3j

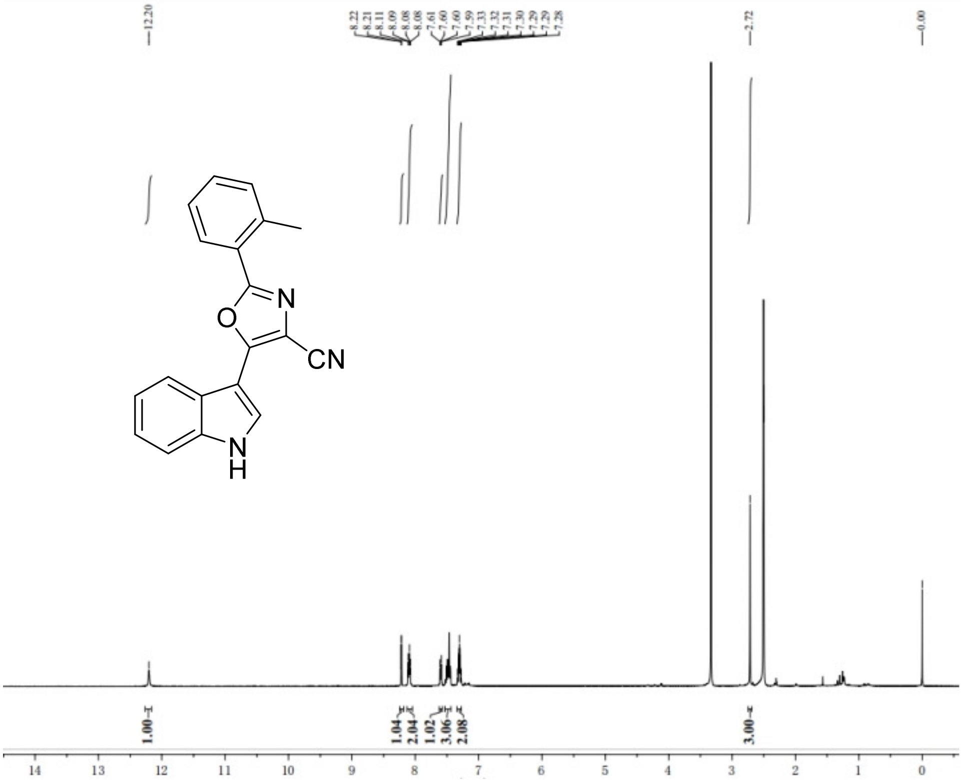

Compound 3k

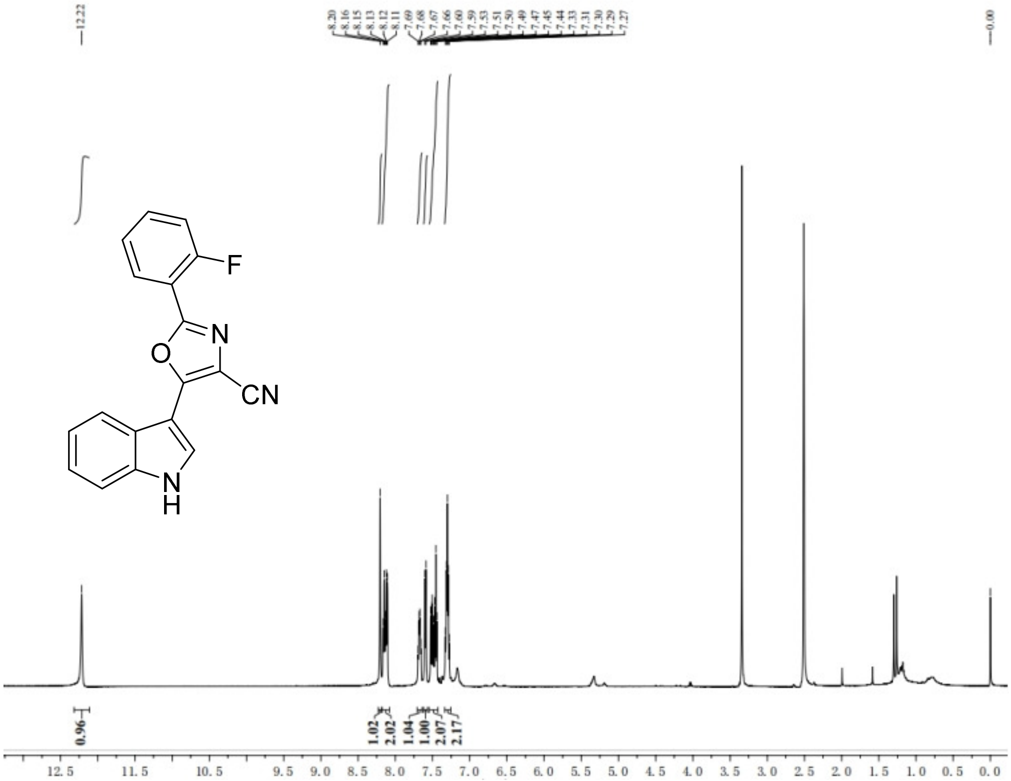

Compound 3l

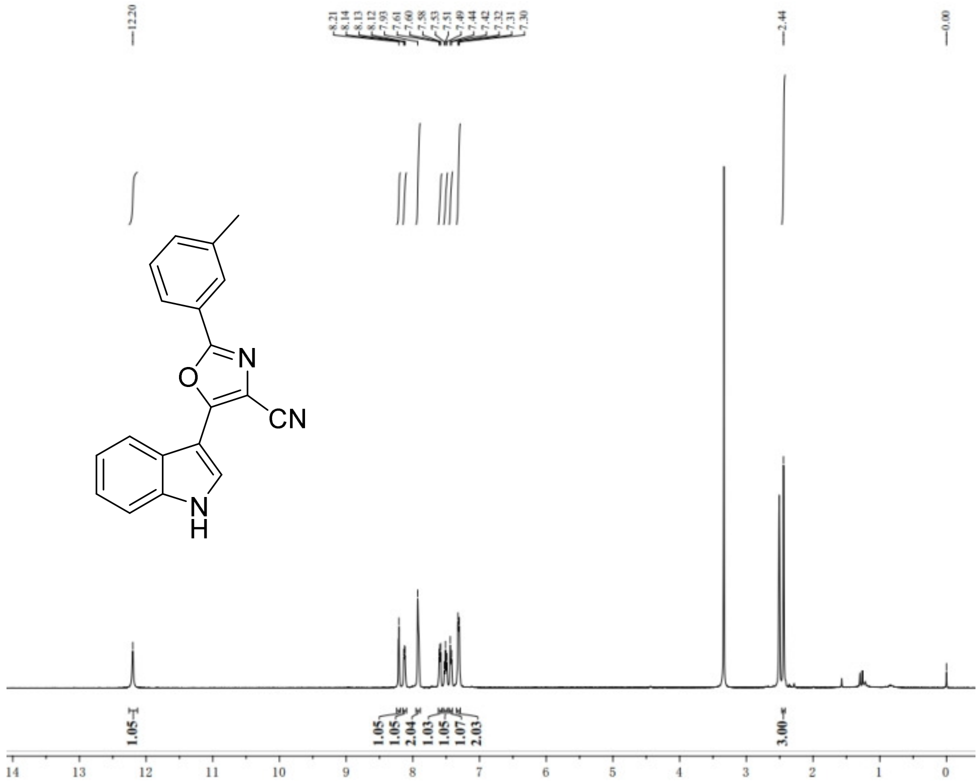

Compound 3m

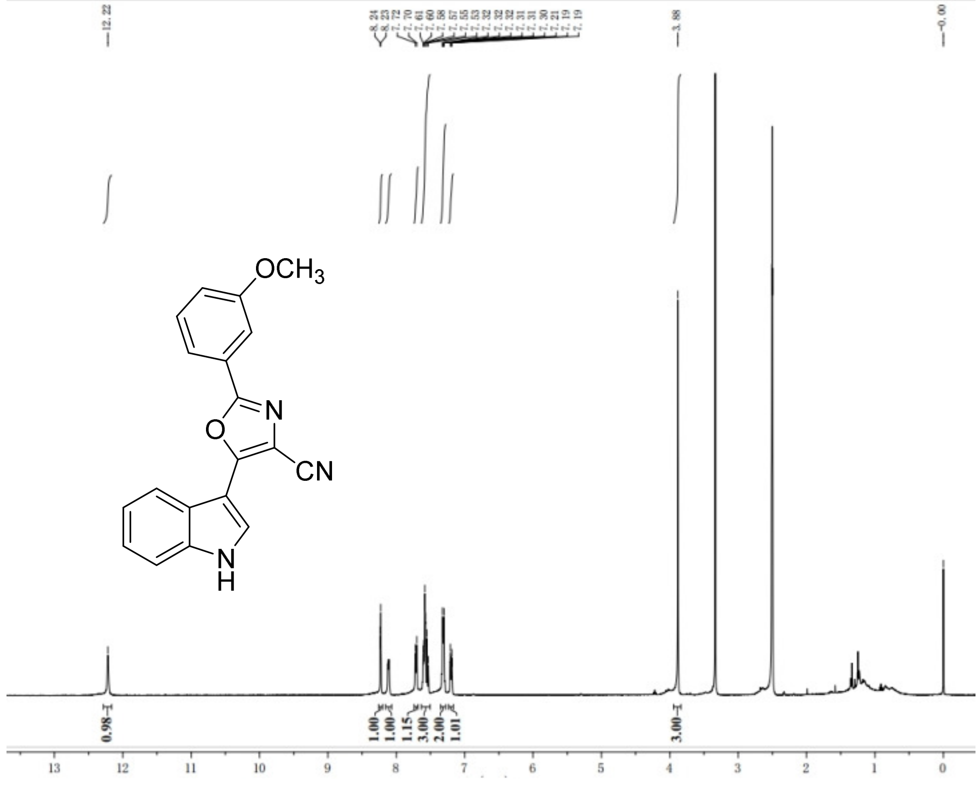

Compound 3n

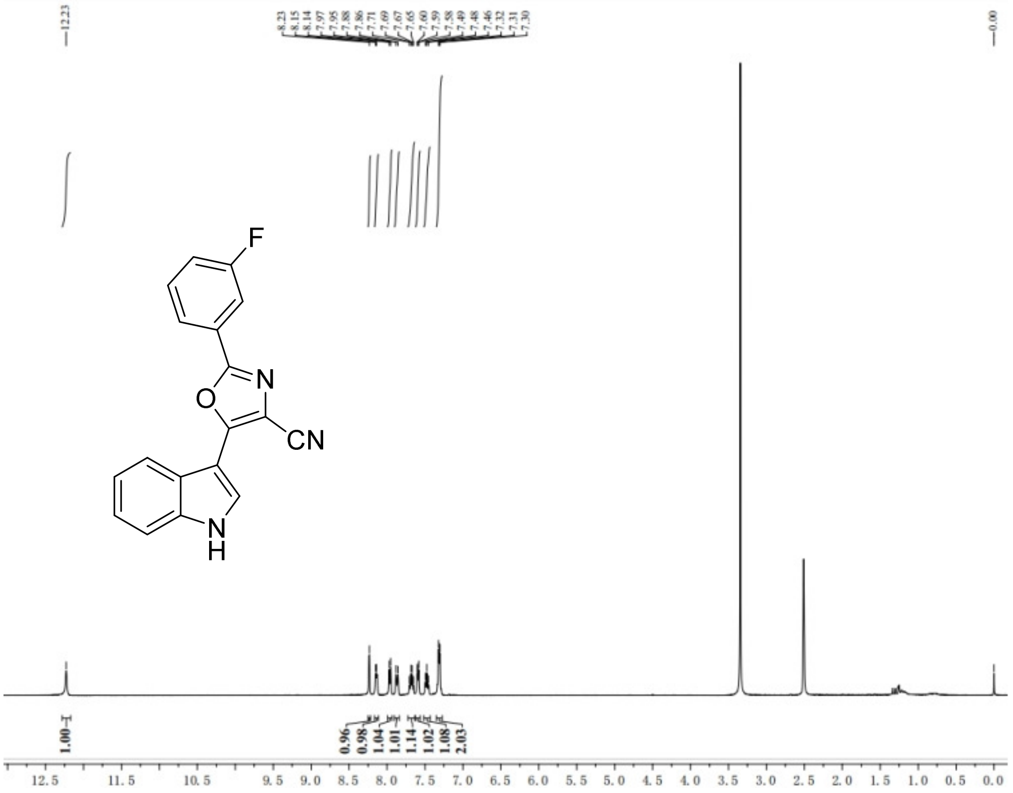

Compound 3o

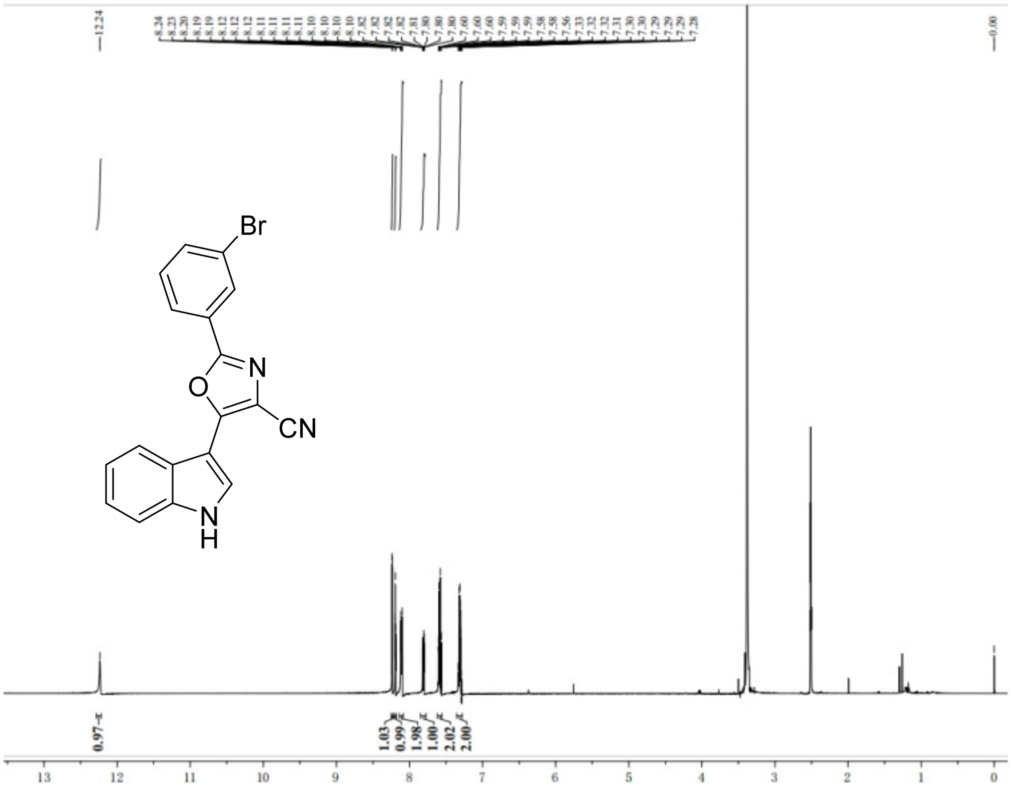

Compound 3p

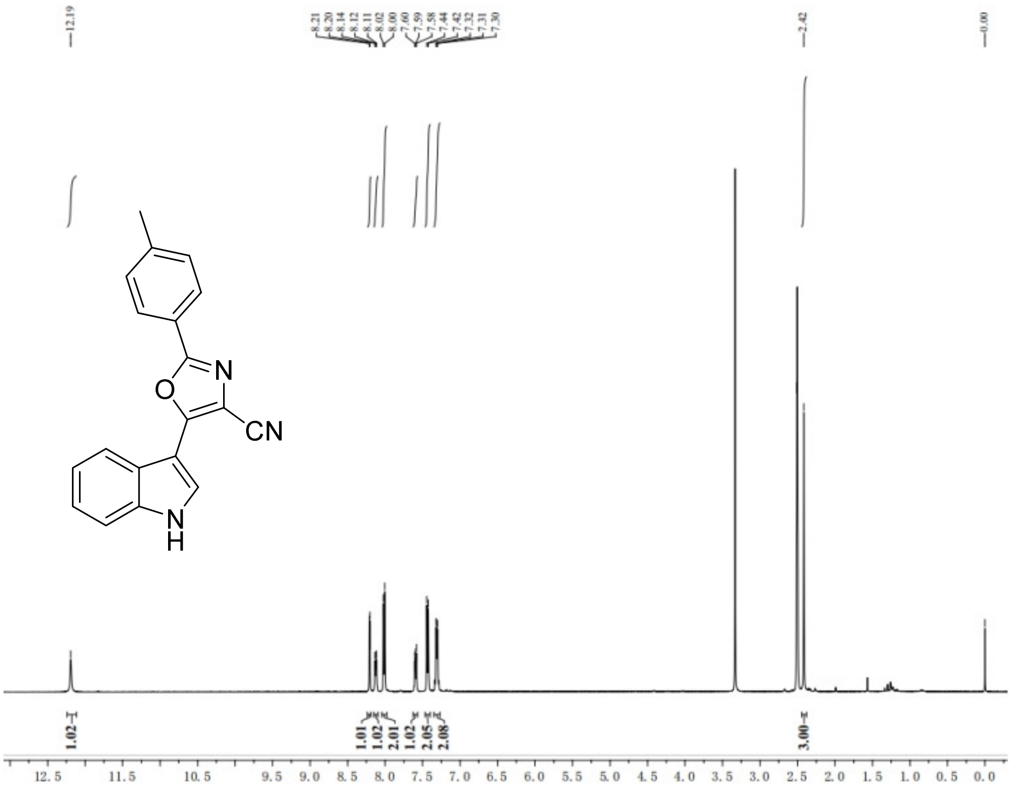

Compound 3q

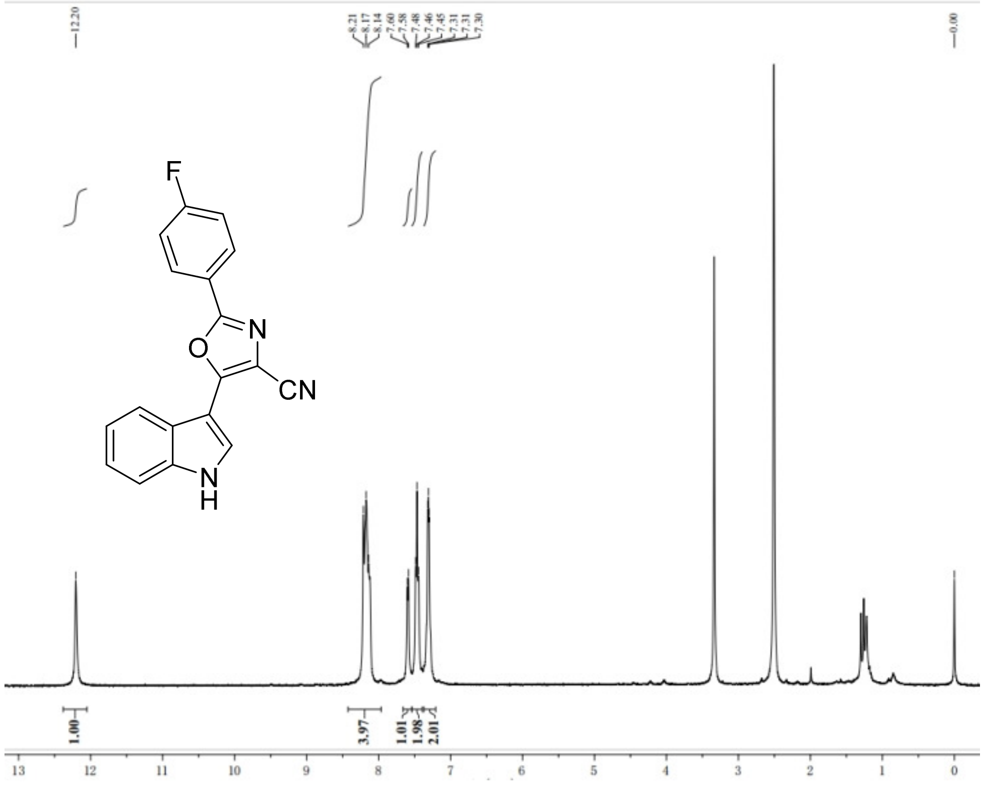

Compound 3r

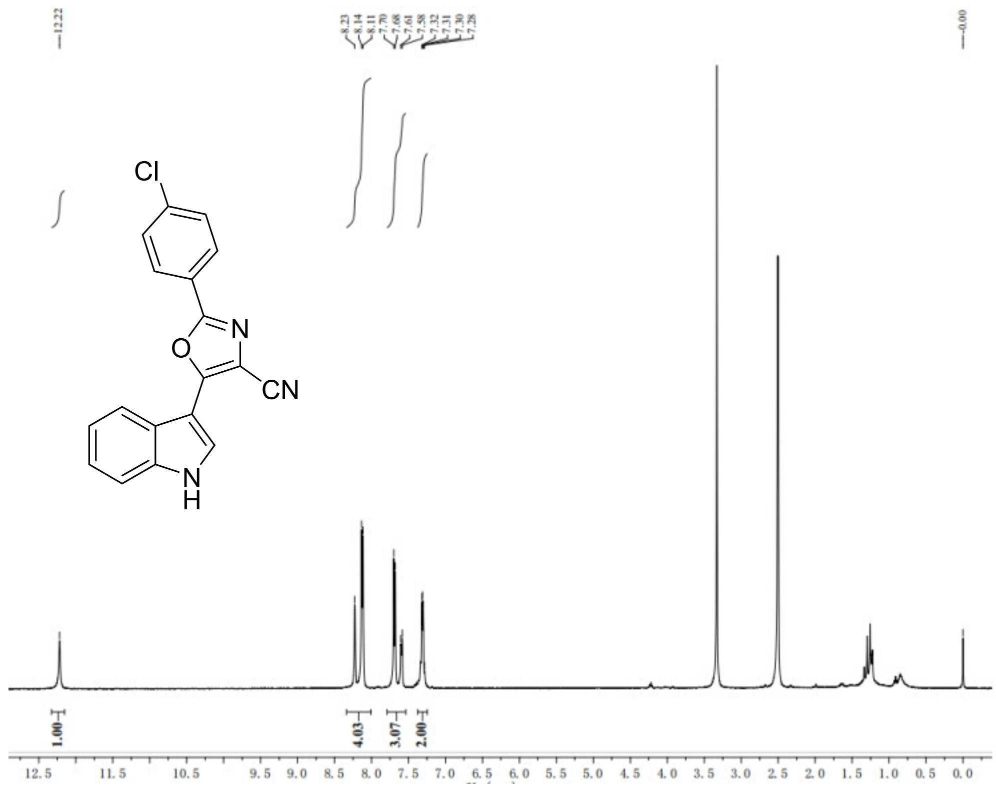

Compound 3s

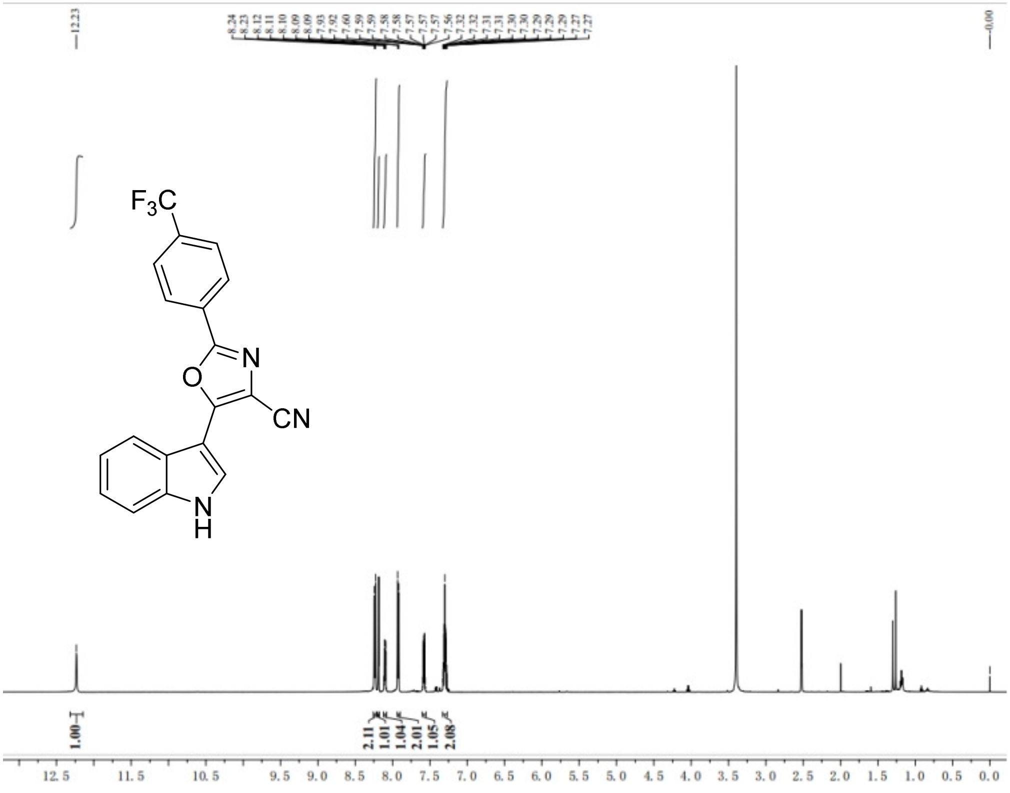

Compound 3t

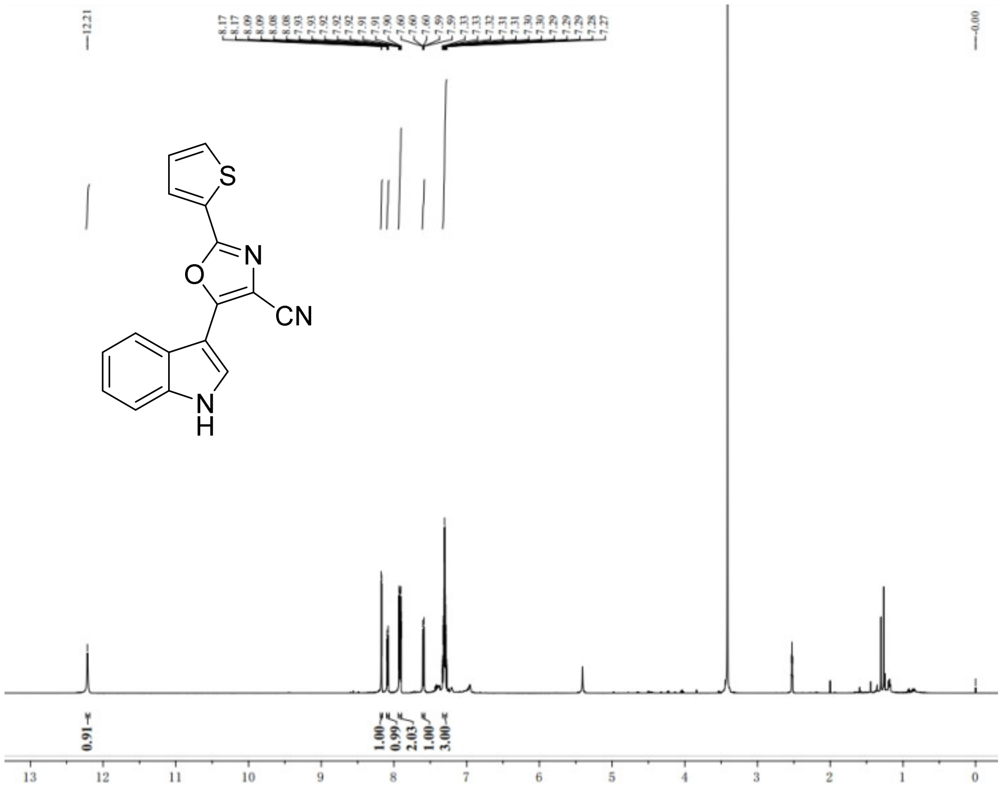
12.21
8.17
8.17
8.09
8.09
8.08
7.93
7.93
7.92
7.92
7.91
7.91
7.90
7.60
7.60
7.60
7.59
7.59
7.33
7.33
7.32
7.31
7.31
7.30
7.30
7.29
7.29
7.28
7.27
0.00
S
N
O
CN
N
H
0.91
1.00
0.99
2.03
1.00
3.00
13
12
11
10
9
8
7
6
5
4
3
2
1
0

2. ^{13}C NMR

Compound 3a

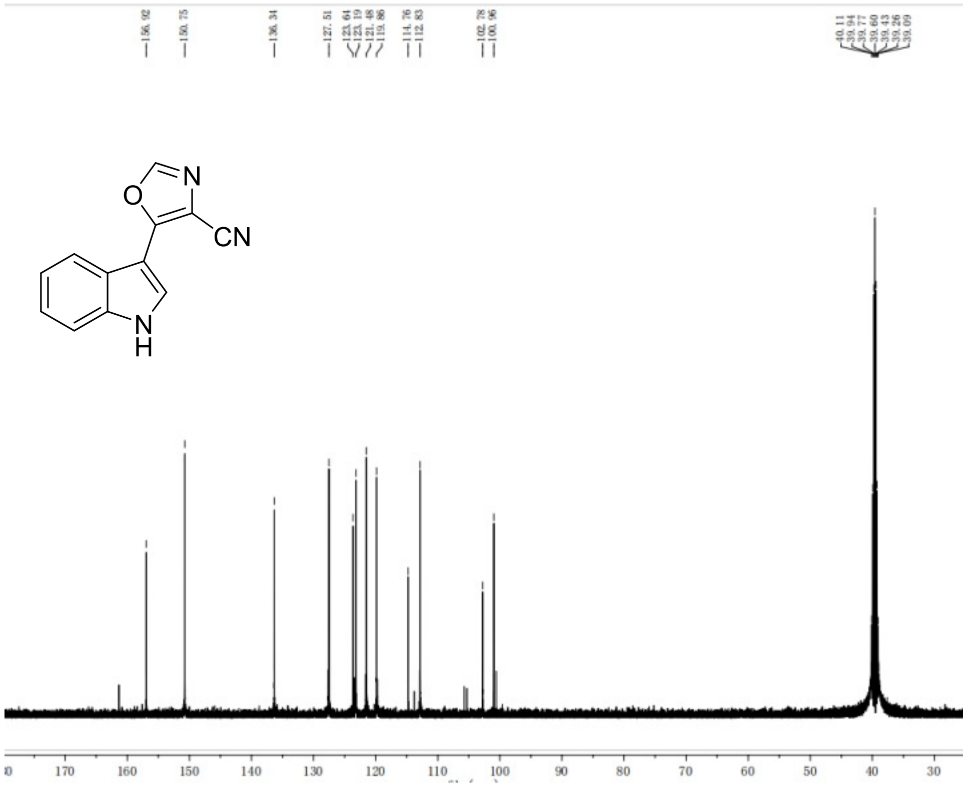

Compound 3b

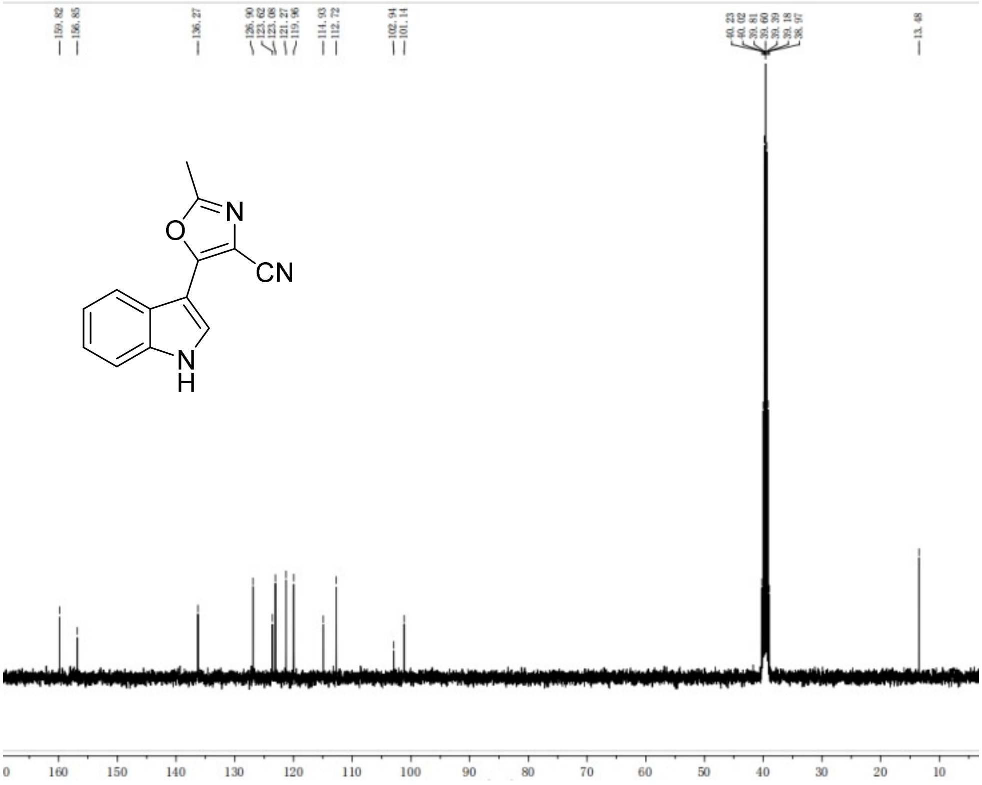

Compound 3c

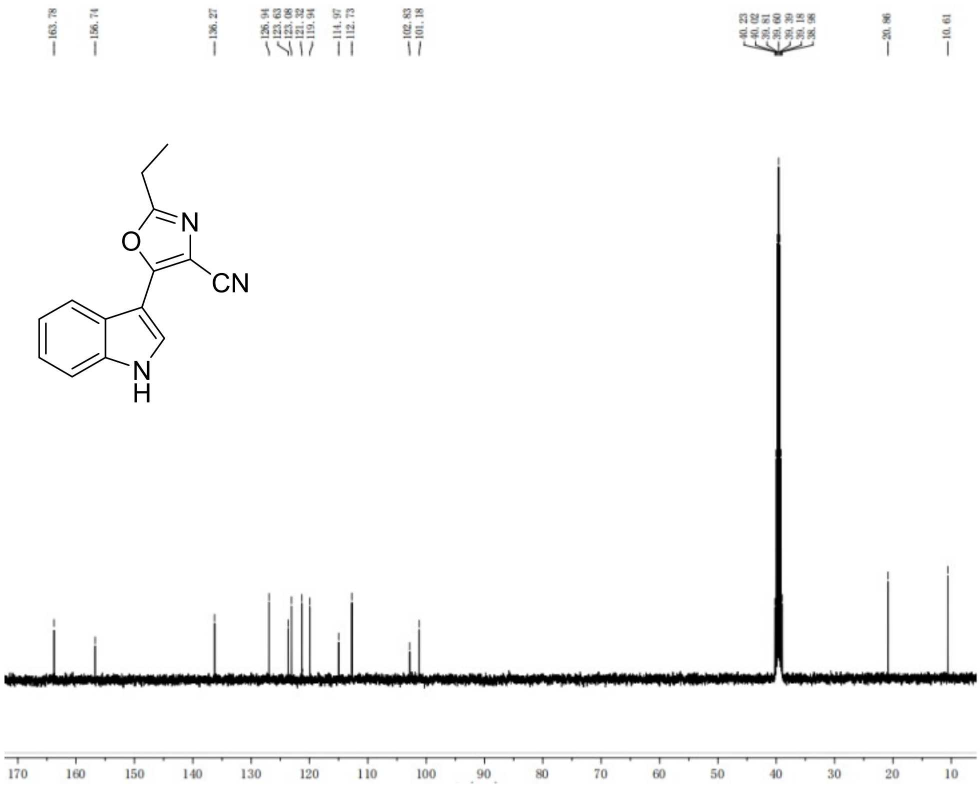

Compound 3d

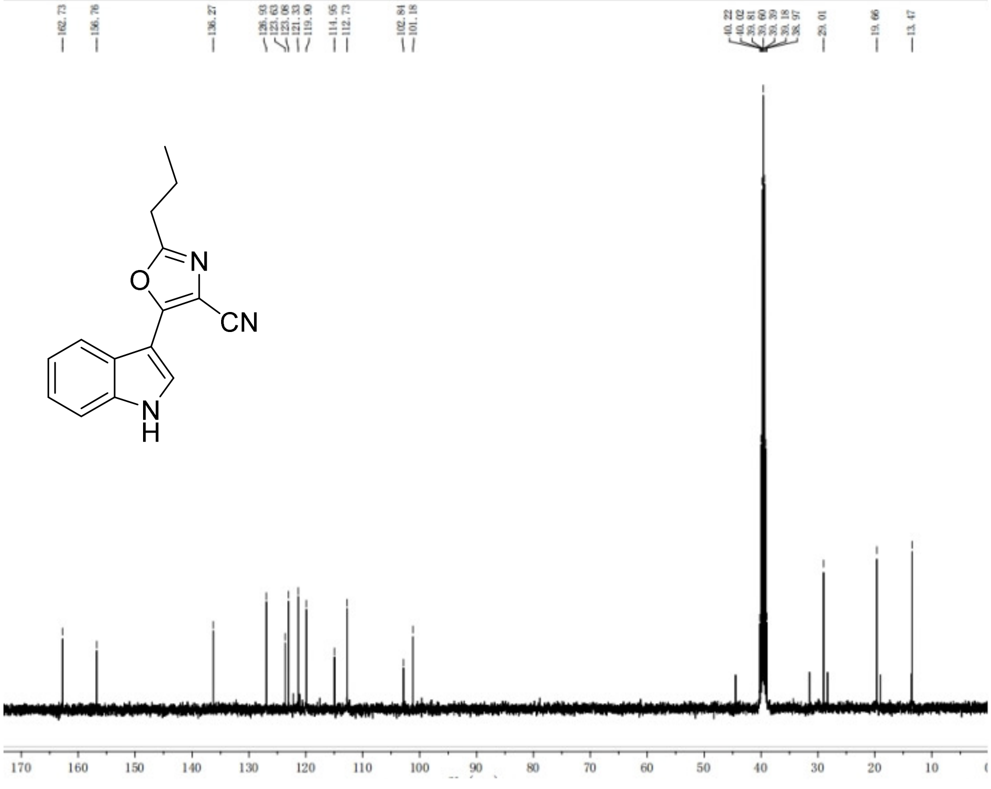

Compound 3e

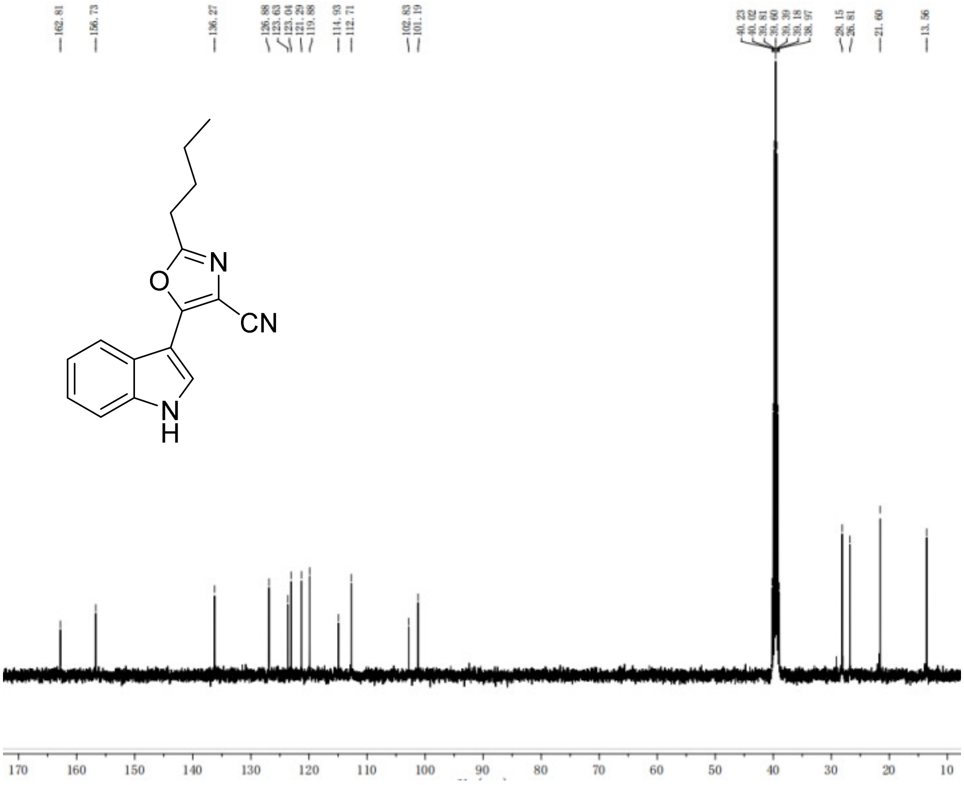

Compound 3f

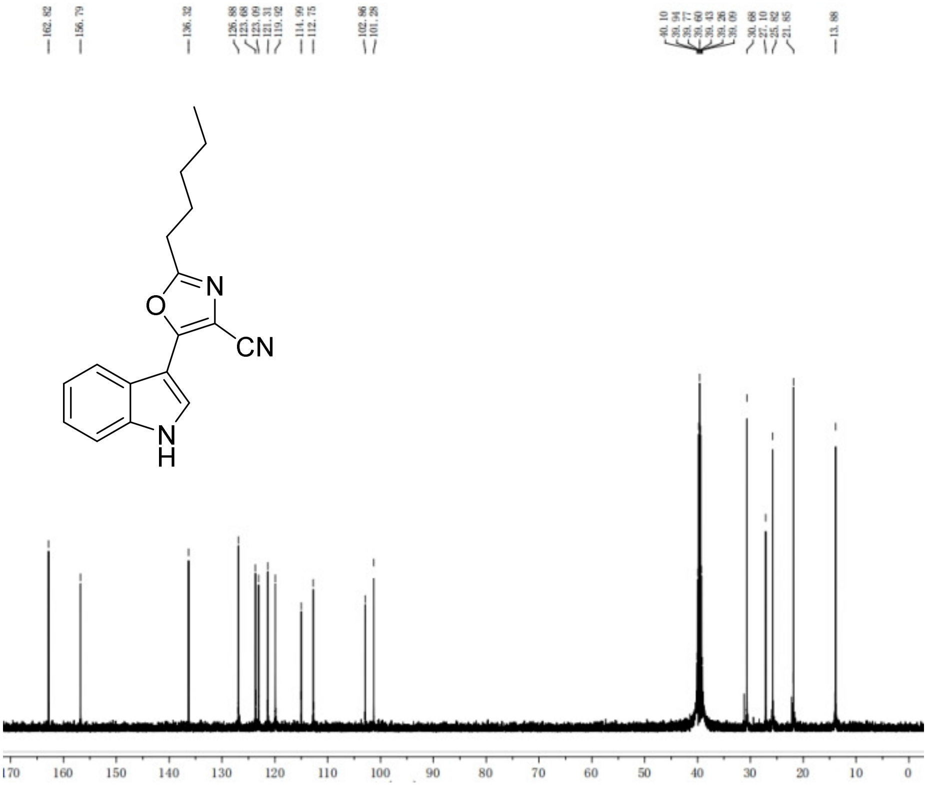

Compound 3g

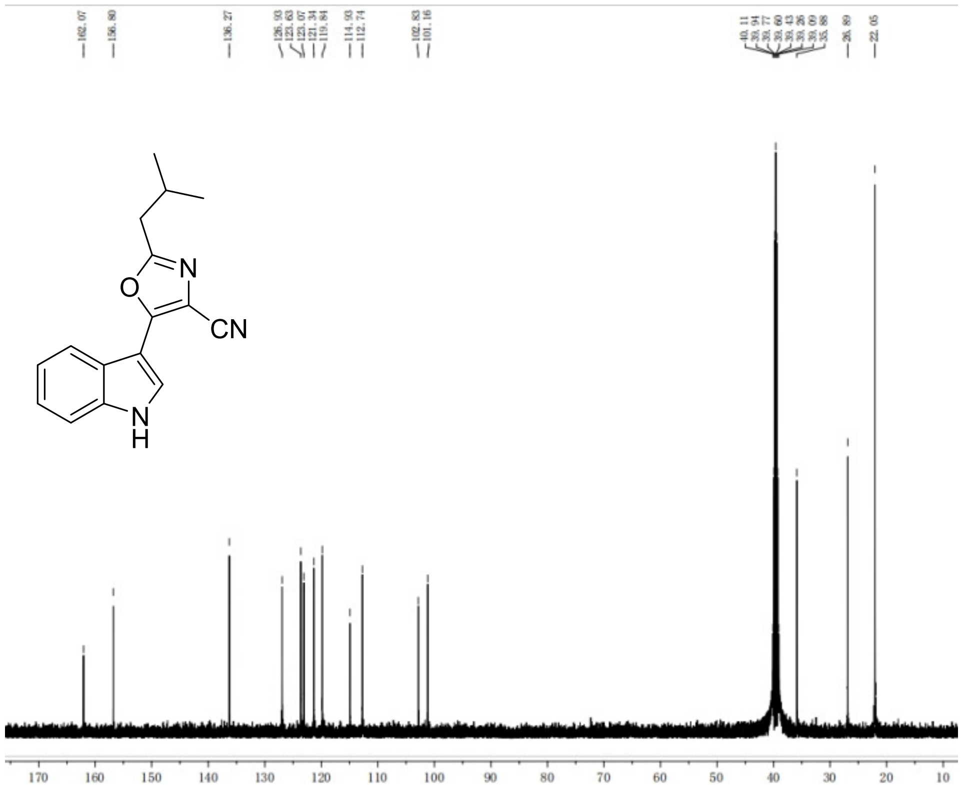

Compound 3h

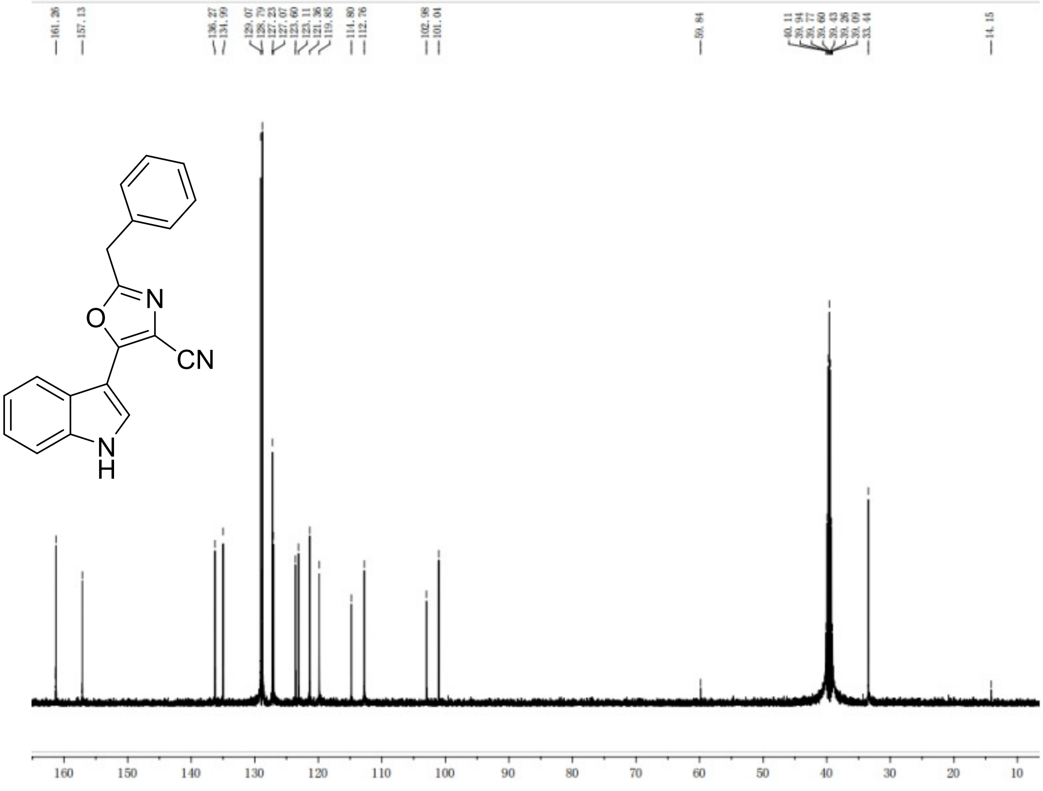

Compound 3i

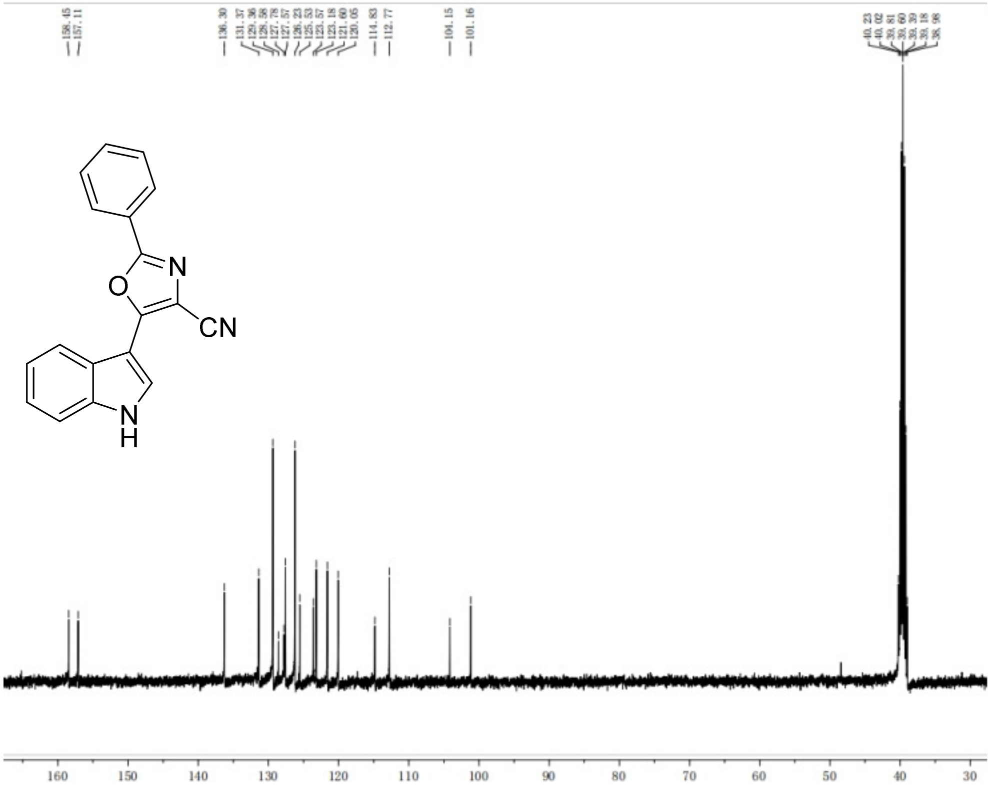

Compound 3j

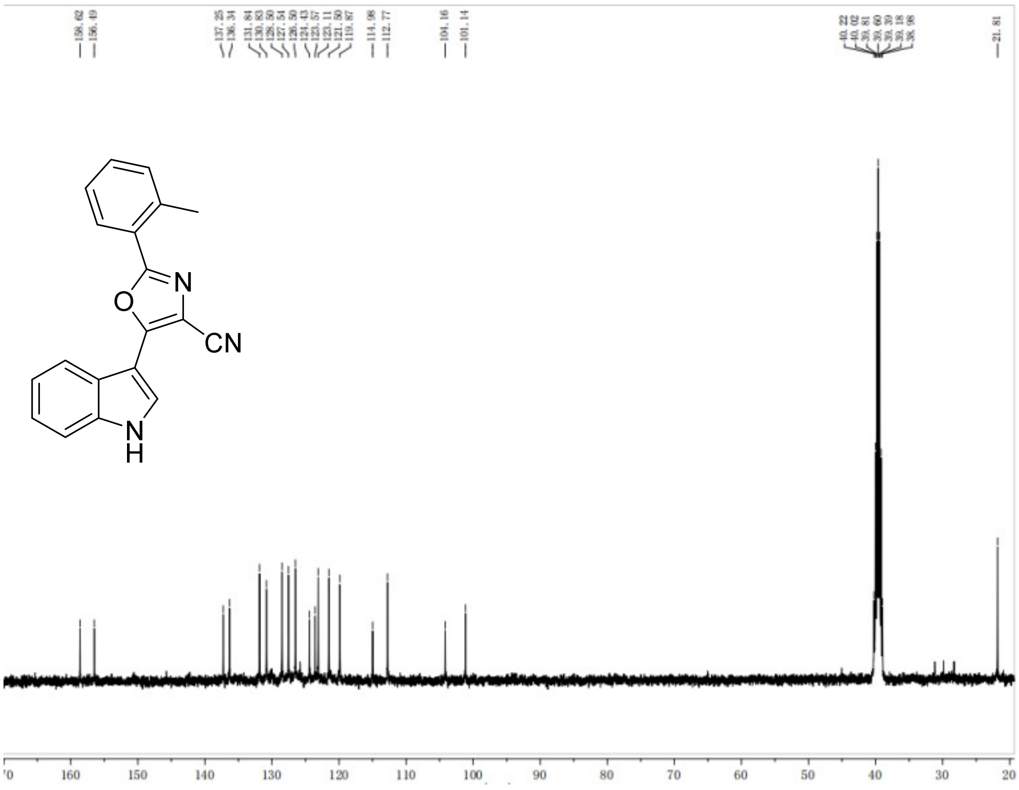

Compound 3k

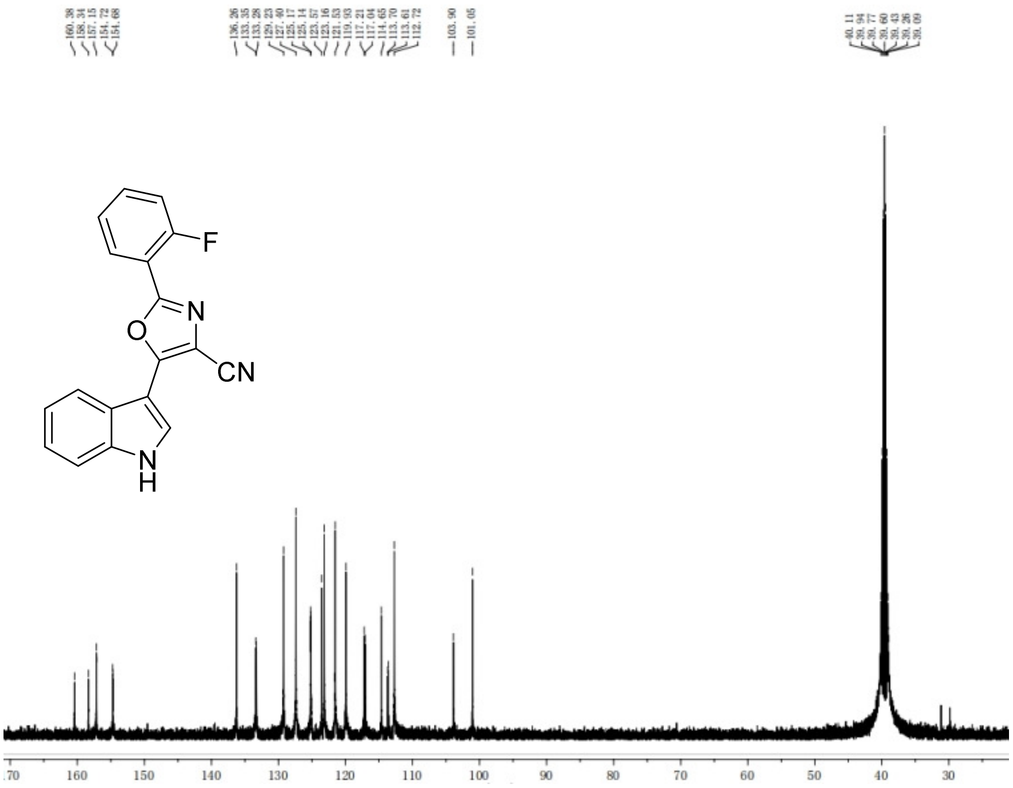

Compound 3l

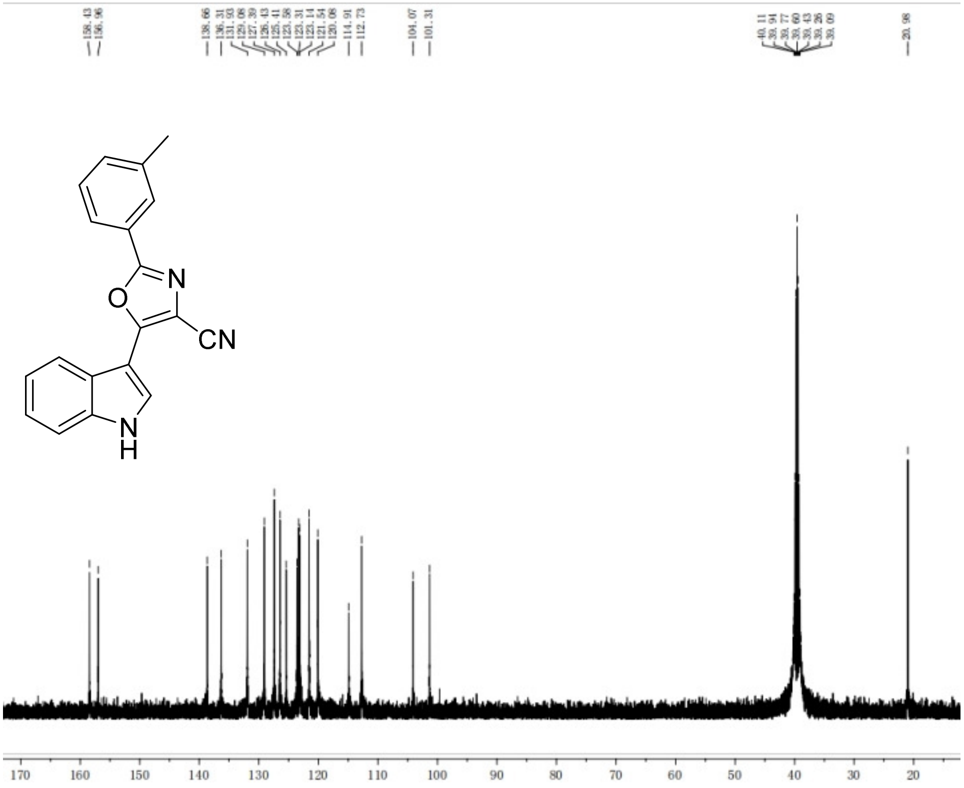

Compound 3m

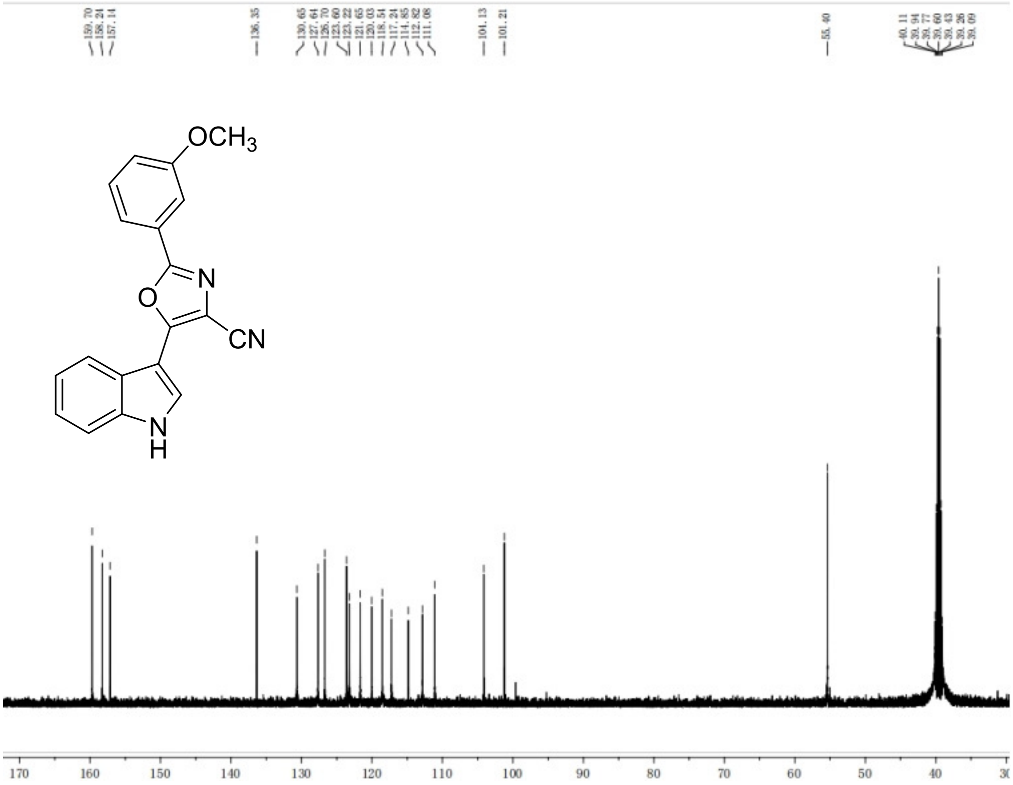

Compound 3n

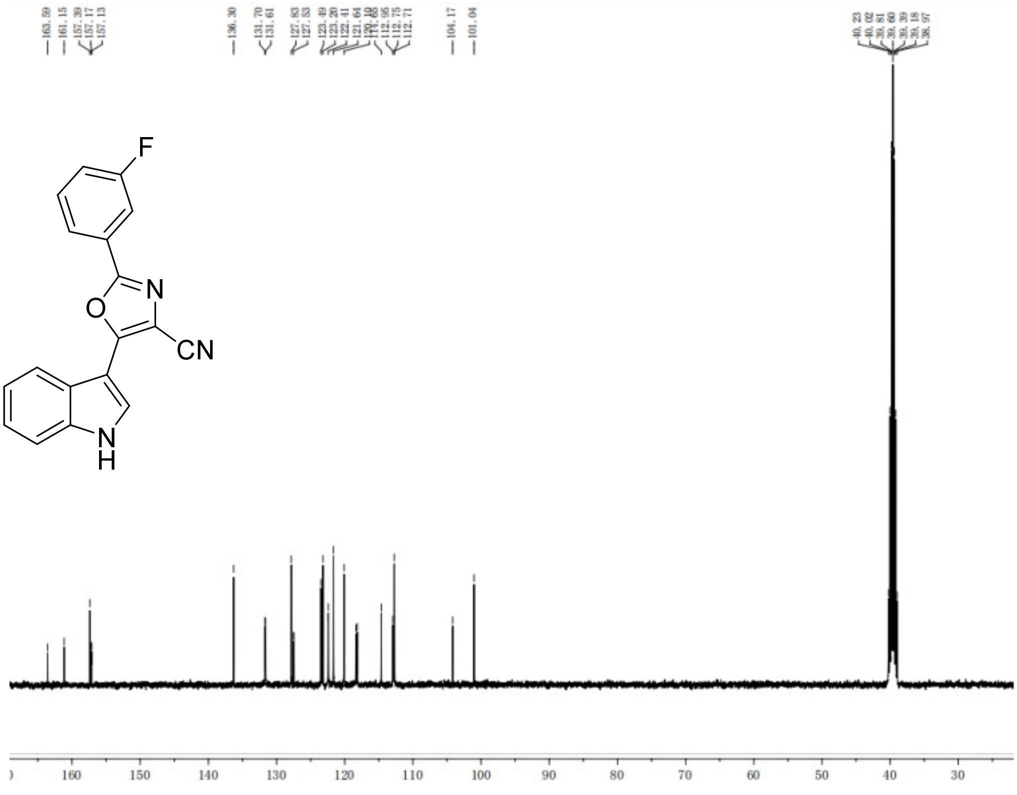

Compound 3o

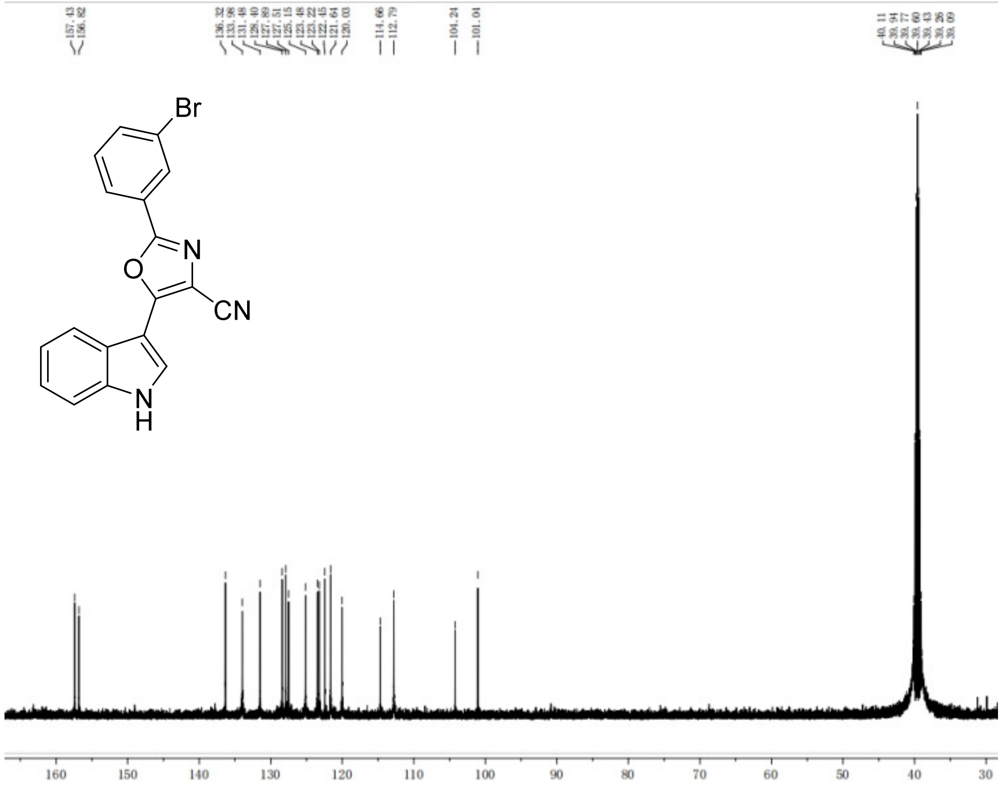

Compound 3p

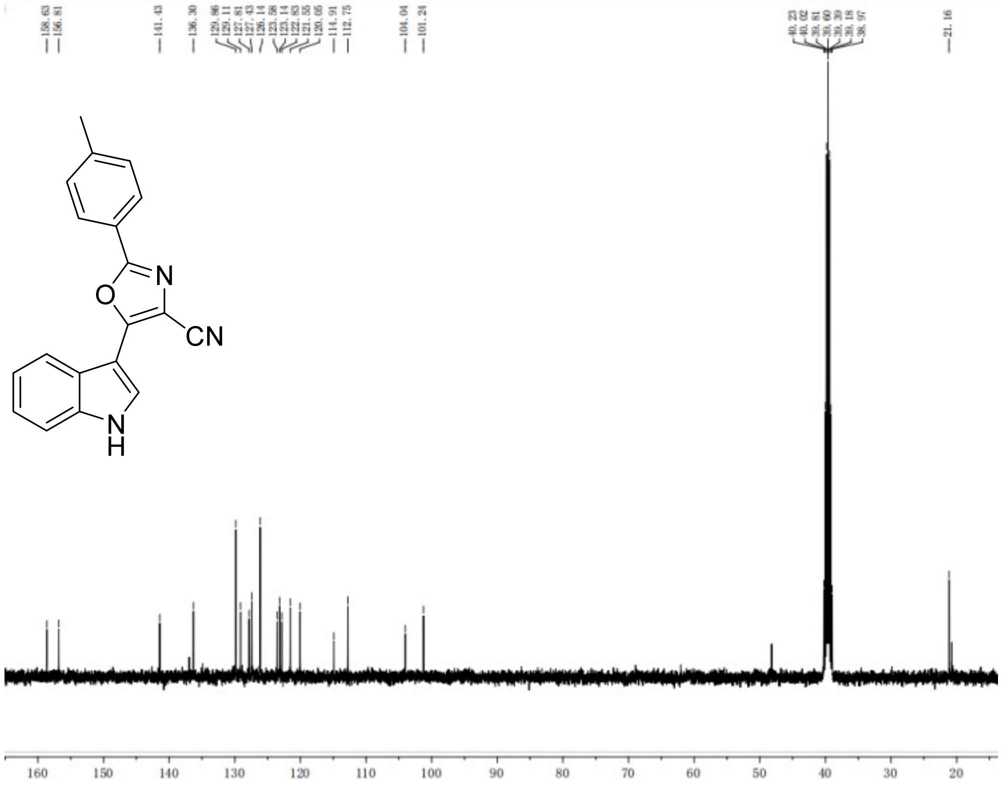

Compound 3q

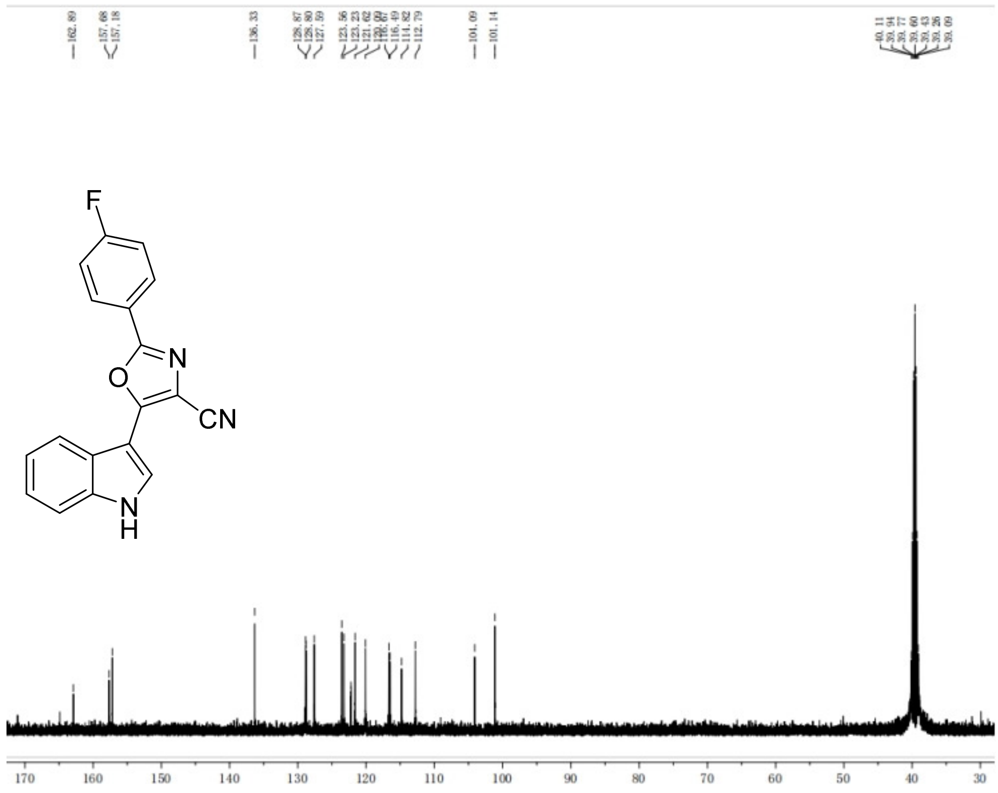

Compound 3r

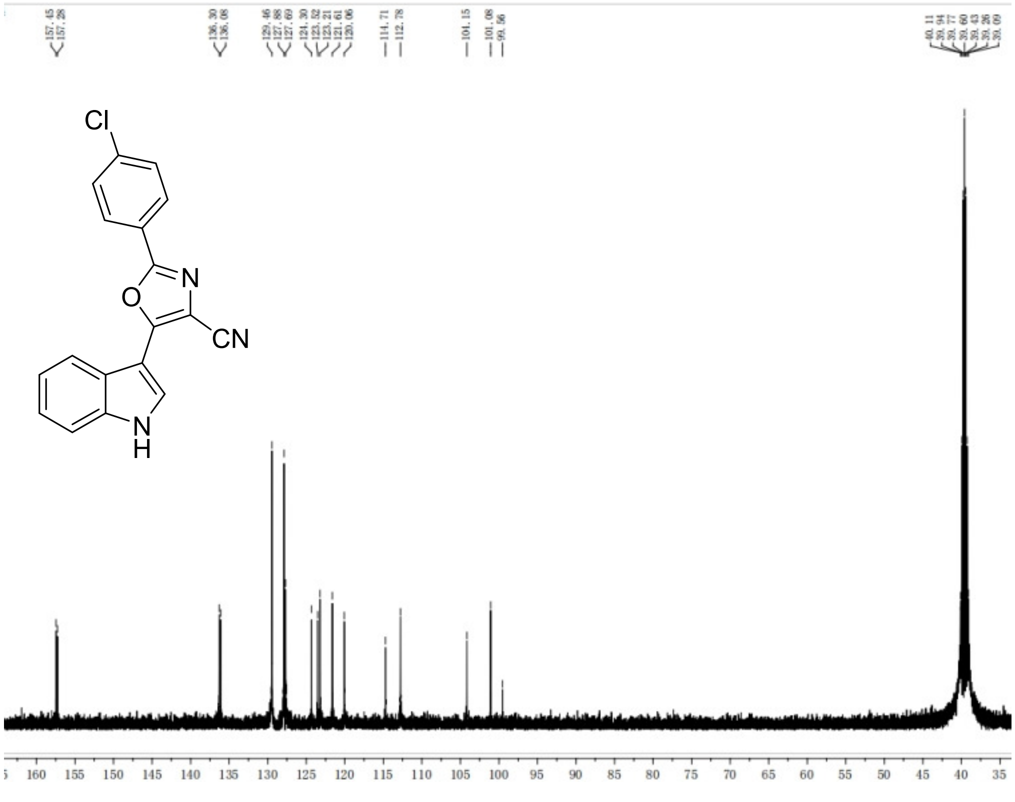

Compound 3s

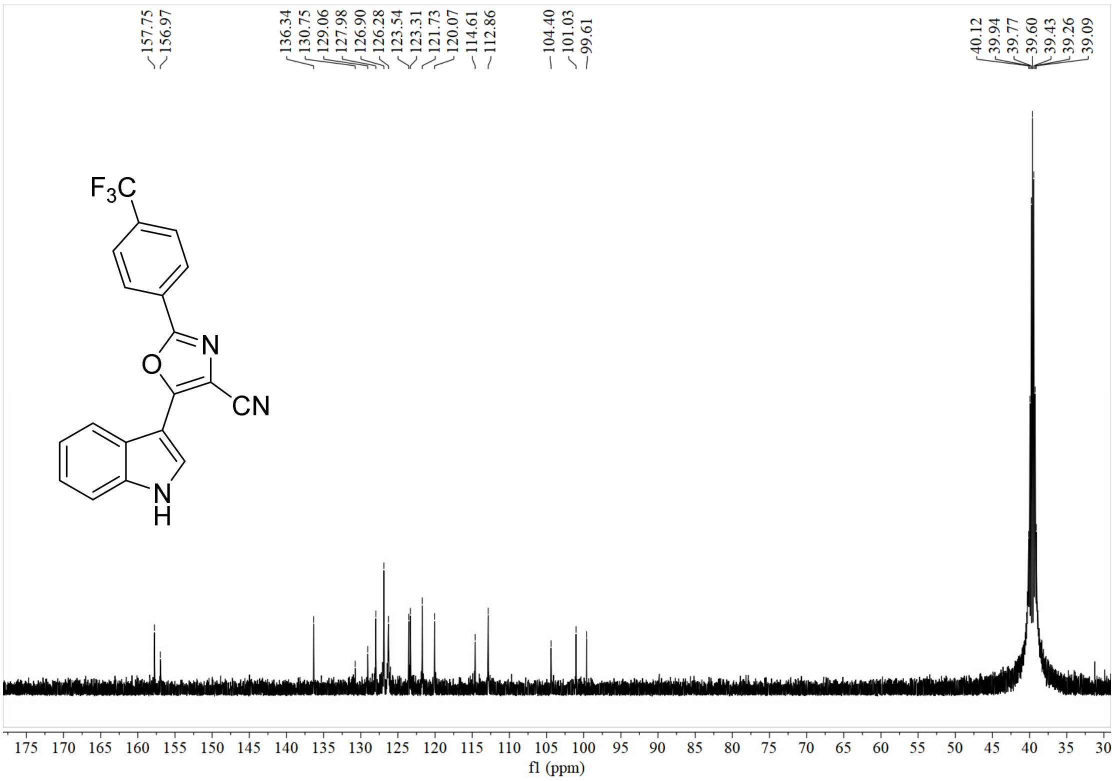

Compound 3t

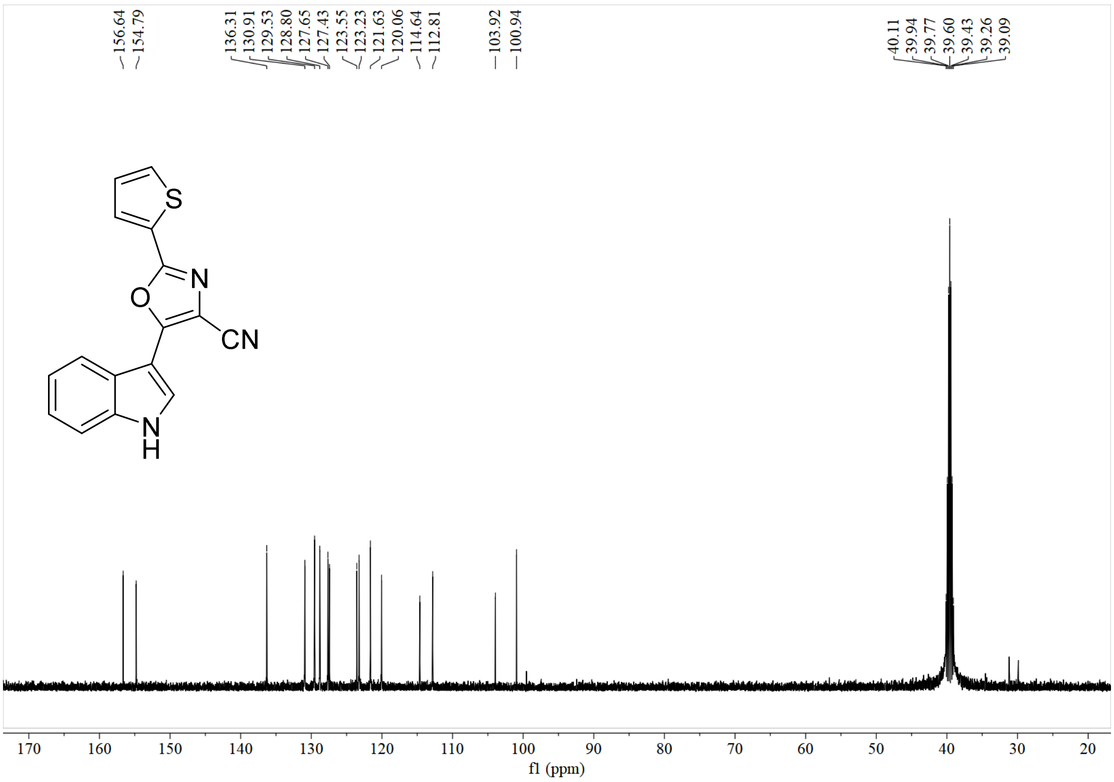

3. HR-MS

Compound 3a

Spectrum from 11.wiff (sample 1) - Sample011, Experiment 1, +TOF MS (100 - 800) from 0.097 to 0.108 min

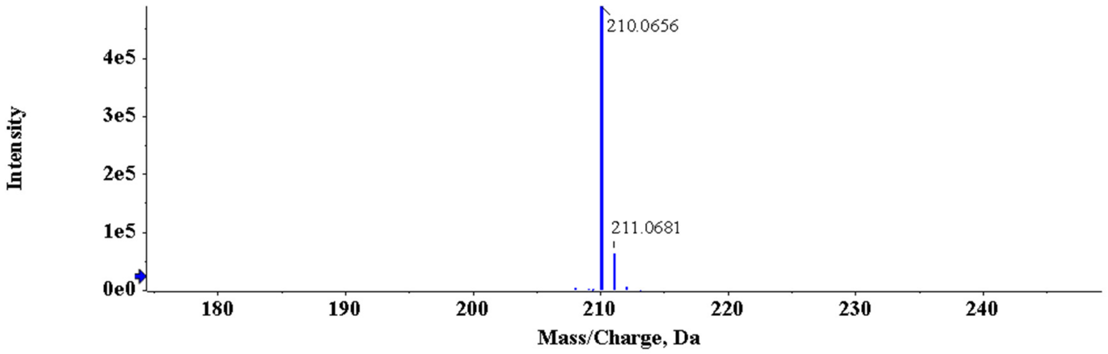

Compound 3b

Spectrum from 09.wiff (sample 1) - Sample009, Experiment 1, +TOF MS (50 - 800) from 0.076 to 0.087 min

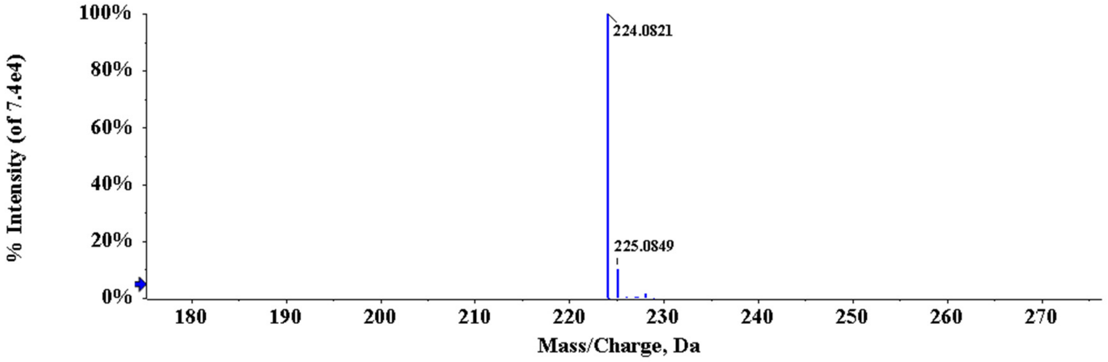

Compound 3c

Spectrum from 06.wiff (sample 1) - Sample006, Experiment 1, +TOF MS (50 - 800) from 0.080 to 0.091 min

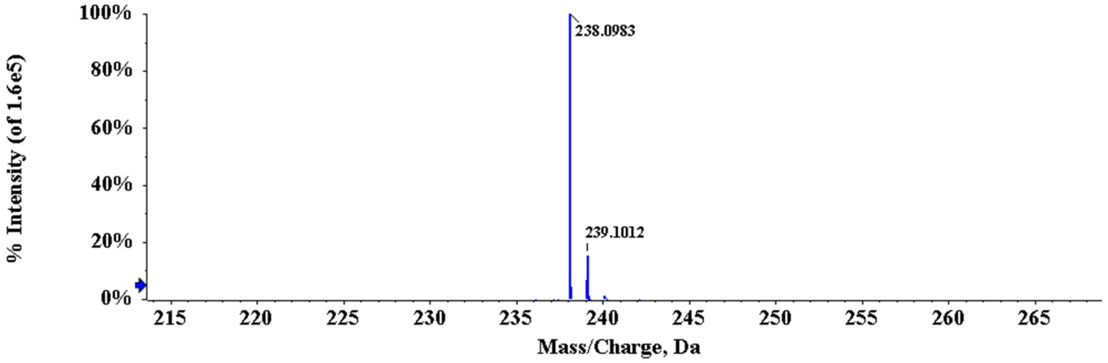

Compound 3d

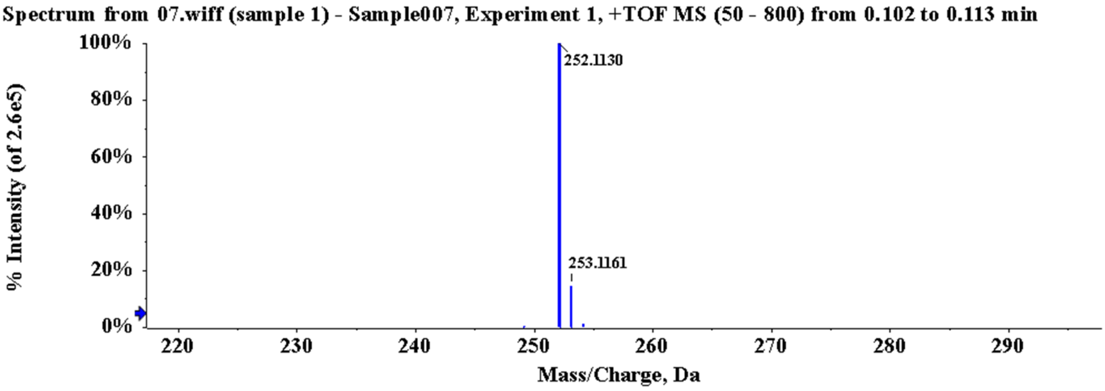

Compound 3e

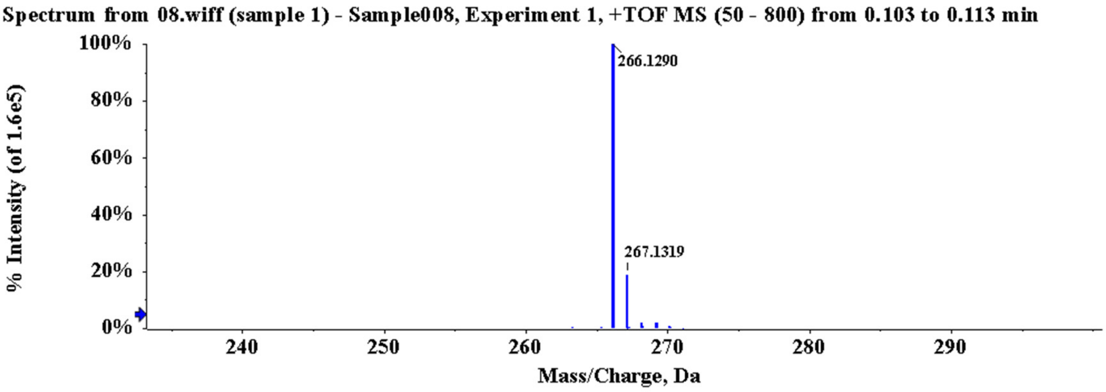

Compound 3f

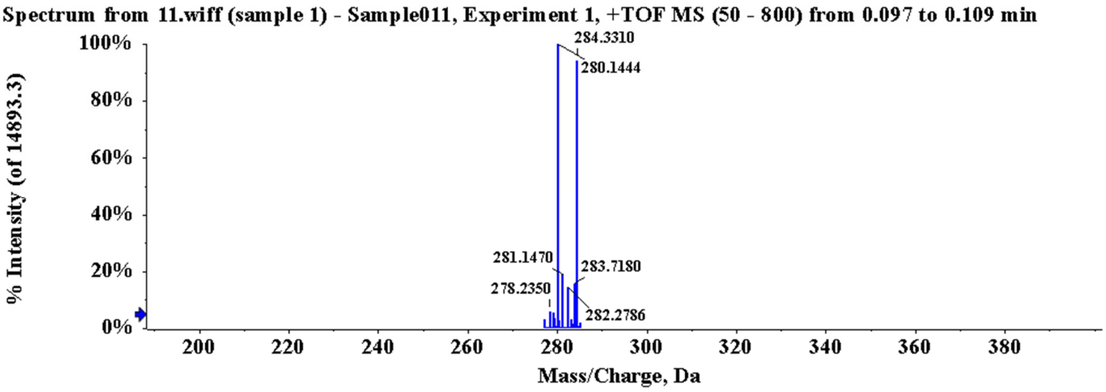

Compound 3g

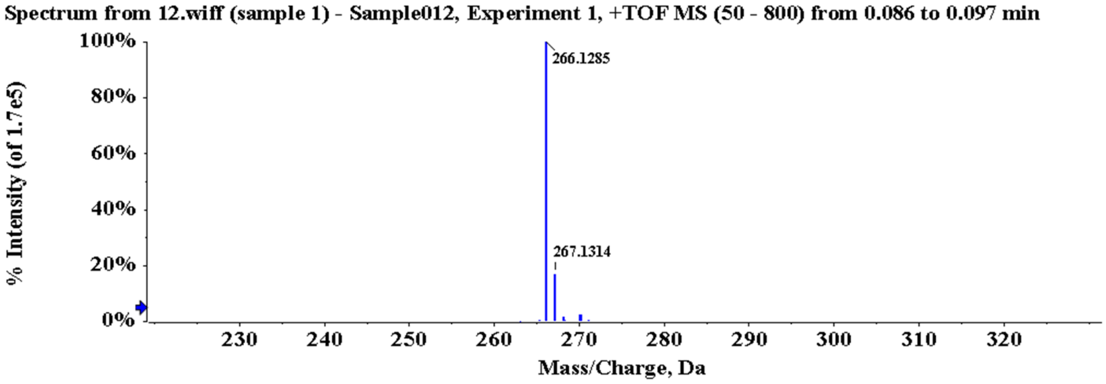

Compound 3h

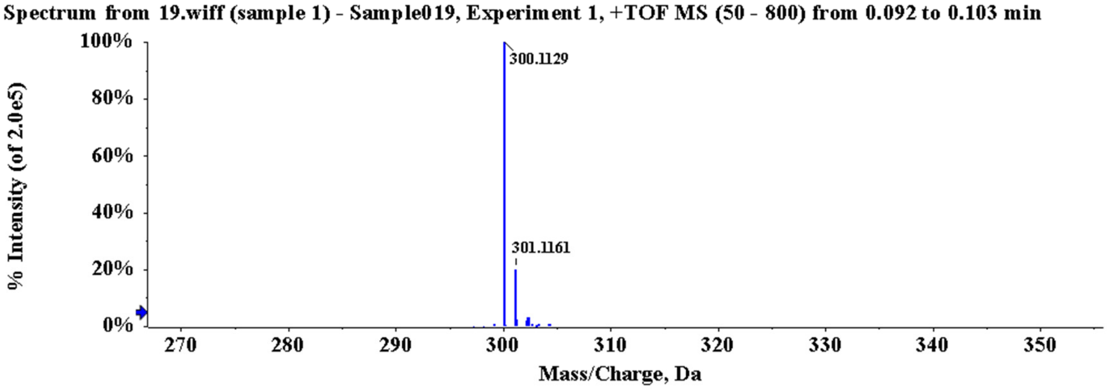

Compound 3i

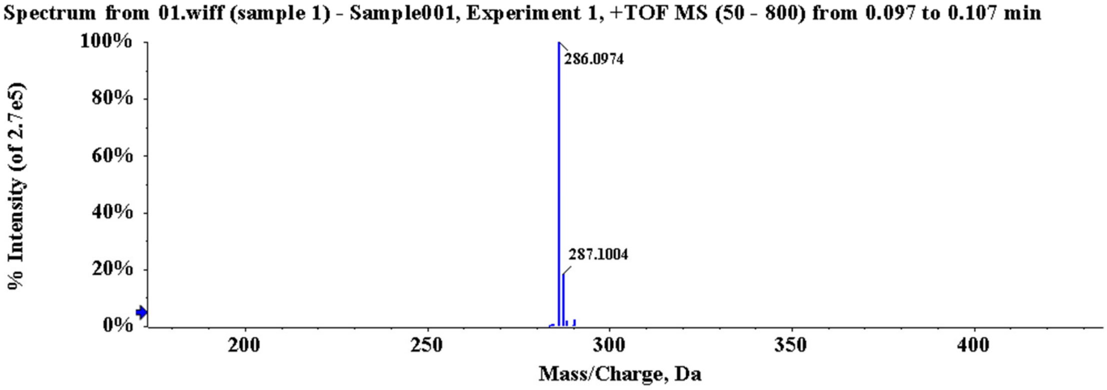

Compound 3j

Spectrum from 05.wiff (sample 1) - Sample005, Experiment 1, +TOF MS (50 - 800) from 0.098 to 0.109 min

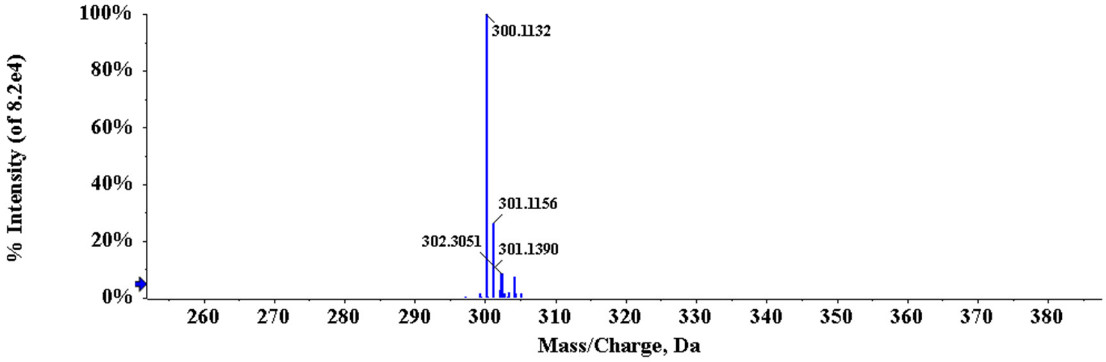

Compound 3k

Spectrum from 18.wiff (sample 1) - Sample018, Experiment 1, +TOF MS (50 - 800) from 0.097 to 0.108 min

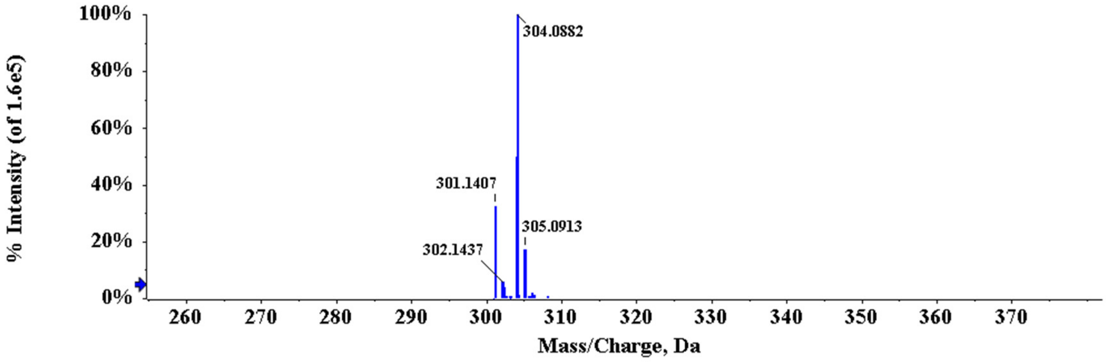

Compound 3l

Spectrum from 03.wiff (sample 1) - Sample003, Experiment 1, +TOF MS (50 - 800) from 0.097 to 0.108 min

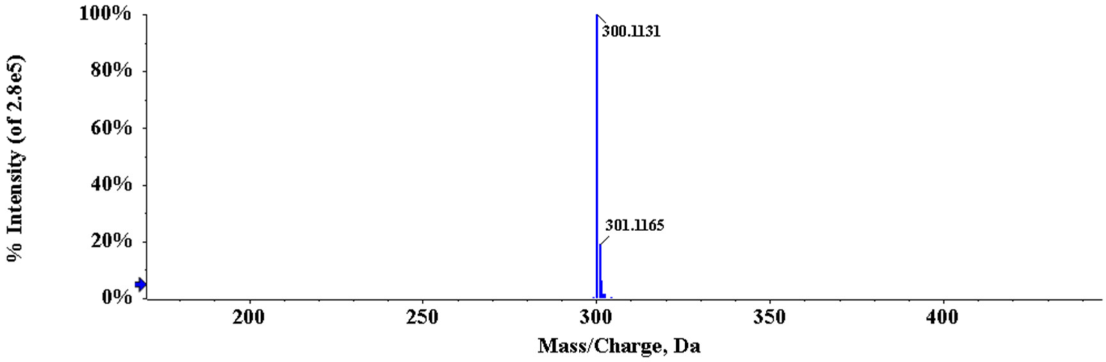

Compound 3m

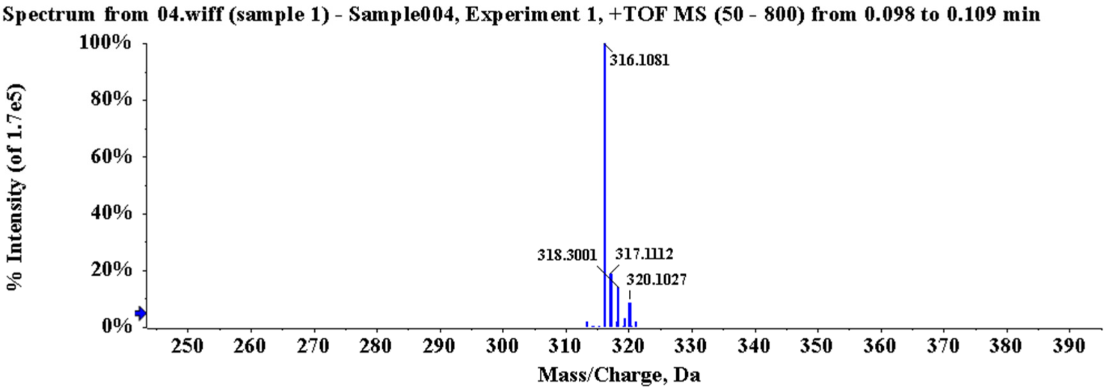

Compound 3n

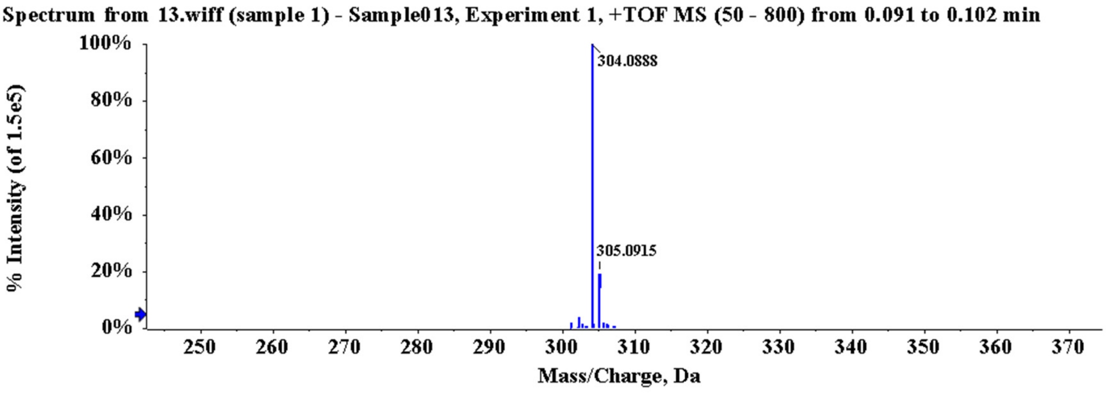

Compound 3o

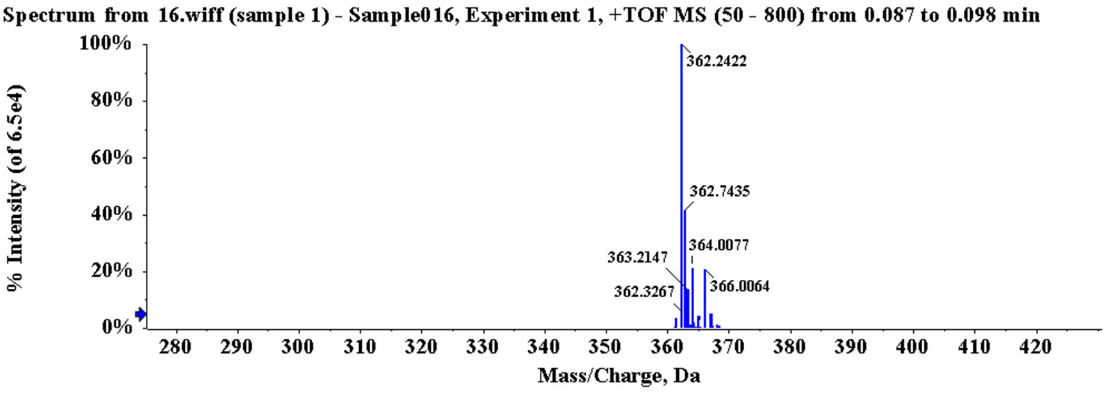

Compound 3p

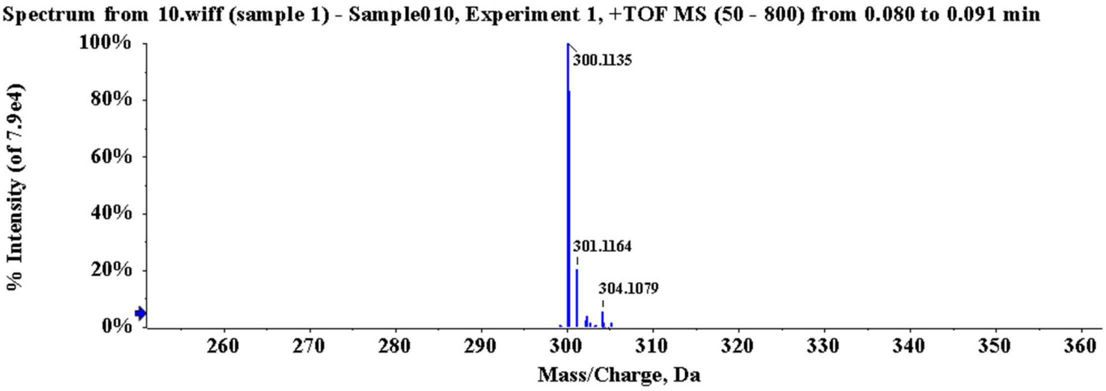

Compound 3q

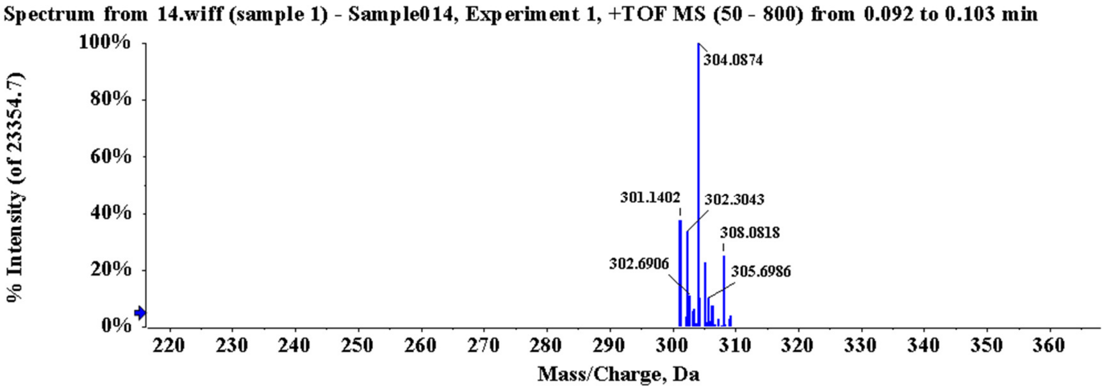

Compound 3r

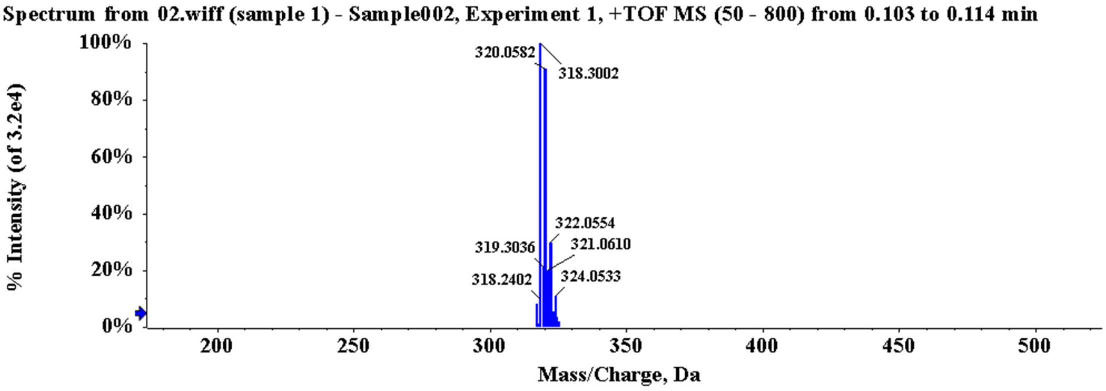

Compound 3s

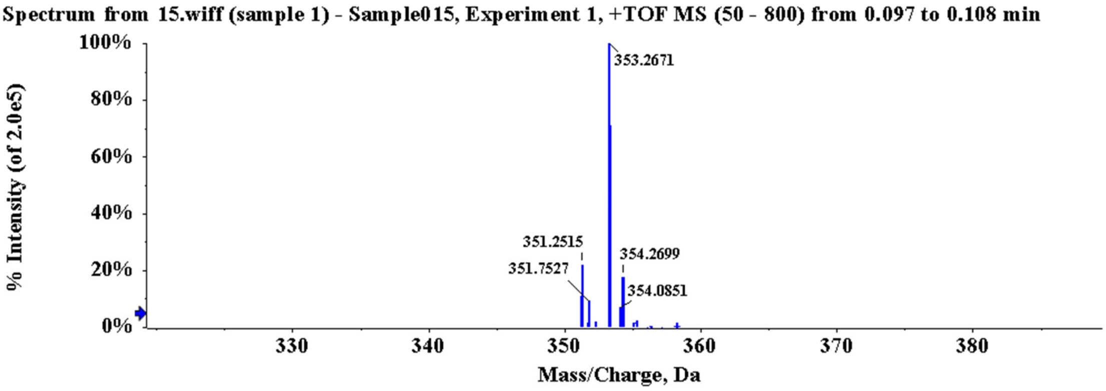

Compound 3t

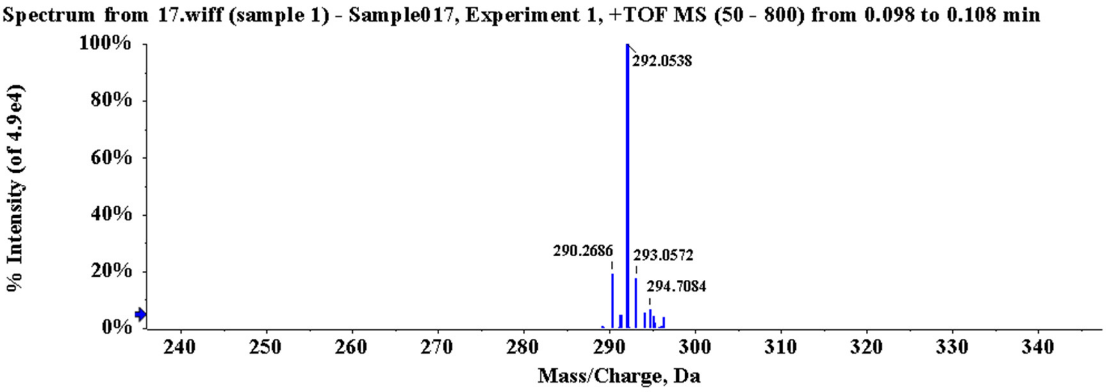

Supplement: Supplementary file 1 [file marinedrugs-21-00103-s001.zip › marinedrugs-2176951-supplementary.pdf]
